# Close spatial and metabolic association between heterotrophic and ammonia-oxidizing marine Nitrososphaerota

**DOI:** 10.1093/ismeco/ycag173

**Published:** 2026-06-18

**Authors:** Qian Li, Mingming Chen, Ye Lu, Cheng Xu, Yue Zheng, Zhirui Zeng, Dapeng Xu, Wei Qin, Yao Zhang

**Affiliations:** State Key Laboratory of Marine Environmental Science, College of Ocean and Earth Sciences, Xiamen University, Xiamen, Fujian 361102, China; State Key Laboratory of Marine Environmental Science, College of Ocean and Earth Sciences, Xiamen University, Xiamen, Fujian 361102, China; State Key Laboratory of Marine Environmental Science, College of Ocean and Earth Sciences, Xiamen University, Xiamen, Fujian 361102, China; State Key Laboratory of Marine Environmental Science, College of Ocean and Earth Sciences, Xiamen University, Xiamen, Fujian 361102, China; Advanced Institute for Marine Studies, Fujian Ocean Innovation Center, Xiamen, Fujian 361102, China; College of the Environment and Ecology, Xiamen University, Xiamen, Fujian 361102, China; Department of Ocean Science and Engineering, Southern University of Science and Technology, Shenzhen, Guangdong 518055, China; State Key Laboratory of Marine Environmental Science, College of Ocean and Earth Sciences, Xiamen University, Xiamen, Fujian 361102, China; Department of Microbiology, Carl R. Woese Institute for Genomic Biology, University of Illinois Urbana-Champaign, Urbana, IL 61801, United States; State Key Laboratory of Marine Environmental Science, College of Ocean and Earth Sciences, Xiamen University, Xiamen, Fujian 361102, China; Advanced Institute for Marine Studies, Fujian Ocean Innovation Center, Xiamen, Fujian 361102, China

**Keywords:** heterotrophic marine Nitrososphaerota, ammonia-oxidizing archaea, global-scale co-occurrence, genomic and metabolic complementarity, vitamin auxotrophy, cellular association, origin and coevolution, horizontal gene transfer

## Abstract

Following the ubiquitous autotrophic ammonia-oxidizing archaea (AOA), heterotrophic representatives of the marine Nitrososphaerota (HMN) form the second most abundant group within this archaeal phylum. However, their eco-evolutionary strategies remain poorly understood. Previous studies have reported a consistent co-occurrence of HMN with marine AOA (MAOA), prompting a detailed investigation into their potential interaction. Through large-scale (meta)genomic and metatranscriptomic analyses, we reveal that HMN possess ultra-streamlined genomes and globally co-occur with marine AOA. The absence of most B vitamin biosynthesis pathways, incomplete citrate cycle and glycolysis, along with the essential requirement for exogenous amino acids, suggest their potential metabolic dependency on AOA. Meanwhile, catalyzed reporter deposition fluorescence in situ hybridization supports a close physical association between HMN and AOA. The nearly synchronous origins of HMN and AOA after oxygen rise, coupled with HMN’s dispersive microhabitats (evidenced by dense, shallow subclades) and extensive horizontal gene transfer between these groups, further support their close relationship—although HMN likely acquired heterotrophic capabilities from bacteria. This study reveals a previously unrecognized association between HMN and AOA, implying a tight coupling between autotrophic and heterotrophic processes in deep-sea habitats.

## Introduction

The phylum Nitrososphaerota (formerly Thaumarchaeota) is an ancient and ubiquitous group of archaea. Its members thrive in remarkably diverse environments, ranging from oceans and freshwater to soils, the subsurface, and even extreme habitats [1–5]. In the ocean, Nitrososphaerota constitute up to 40% of the total prokaryoplankton in the mesopelagic and bathypelagic zones [6], playing a vital ecological role. Within Nitrososphaerota, the most well-known are the chemolithoautotrophic ammonia-oxidizing archaea (AOA), which are responsible for the rate-limiting first step of nitrification and significantly impact marine carbon and nitrogen cycles [7]. In addition to AOA, there are non-ammonia-oxidizing, putative aerobic heterotrophic Nitrososphaerota in the ocean. The heterotrophic marine Nitrososphaerota (HMN, also known as pSL12-like lineage) were initially identified in the North Pacific Subtropical Gyre through the clustering of archaeal SSU rRNA genes [8], which are highly similar to the clone sequence of pSL12 derived from the Yellowstone hot spring [9]. While HMN are less abundant than AOA (with abundances reaching up to 6% of AOA) [10], they are widely distributed, ranging from nearshore to open-sea habitats and from the surface to the deep sea [11–15].

To date, HMN remain uncultured, but limited metagenome-assembled genomic data indicate heterotrophic metabolic capabilities, including pyrroloquinoline quinone (PQQ)-dependent oxidation of alcohol or sugar, metabolic pathways for fatty acids, amino acids, and methanol, along with anaplerotic CO_2_ assimilation mediated by ribulose-bisphosphate carboxylase (RuBisCO) [10, 16]. In addition, HMN were reported to occupy a distinctive evolutionary origin node on the phylogenetic tree of Nitrososphaerota, completely distinct from the deeply-rooted heterotrophic Nitrososphaerota derived from terrestrial environments [10].

An intriguing observation emerged from early studies, which suggested significant co-occurrence between the SSU rRNA gene of HMN and the archaeal ammonia monooxygenase (*amoA*) gene [13, 17, 18]. However, assembled genomic analyses so far did not recover *amoA* gene in HMN genomes [10, 16]. This context prompted us to explore the possible association between HMN and marine AOA, raising questions about the existence of a (facultative) prokaryotic symbiotic partner. However, the limited metabolic, ecological, and evolutionary insights into HMN constrain our exploration of these questions.

To bridge these knowledge gaps, we conducted comprehensive analyses that integrated all available 104 HMN genomes, over 2500 genomes from AOA and other typical marine microorganisms, as well as extensive metagenomic and metatranscriptomic datasets encompassing: (i) this study (the South China Sea and Western Pacific), (ii) *Tara Oceans* and *Malaspina* expeditions, and (iii) public repositories and published studies. These analyses—complemented by catalyzed reporter deposition fluorescence in situ hybridization (CARD-FISH)—reveal overlapping ecological niches between HMN and MAOA, their reciprocal metabolic coupling, and coordinated evolutionary trajectories. Together, they support a spatial and metabolic association between deep-sea-dwelling HMN and MAOA.

## Materials and methods

### Detailed methods are provided in supplementary information.

#### Sample collection and DNA extraction

A total of 67 seawater metagenomic samples were collected from 11 stations in the South China Sea (SCS) and the Western Pacific during six cruises between 2019 and 2022 (Table S1), with environmental parameters measured using a conductivity–temperature–depth (CTD) system. Seawater (20–570 L) was sequentially size-fractionated by filtration through membranes with pore sizes of 10 μm, 3/1.6/0.8 μm, and 0.3/0.2 μm (142 mm diameter; polycarbonate or glass fiber filters, Millipore and Advantec, respectively), and DNA was extracted using the phenol-chloroform-isoamyl alcohol method [19].

#### Metagenome sequencing, co-assembly, and binning

Metagenomic libraries were constructed and sequenced using an Illumina NovaSeq 6000 platform using paired-end (2 × 150 bp) sequencing. Raw reads were quality-controlled with fastp v0.20.0 [20] and subsequently co-assembled by Megahit v1.2.9 [21], grouped according to sampling location, depth, and size fraction. Metagenome-assembled genomes (MAGs) were generated using Anvi’o v7, dereplicated with dRep v3.4.2 [22], and assessed for quality using CheckM v1.1.3 [23]. Taxonomic classification was performed with GTDB-Tk [24]. This pipeline yielded three high-quality heterotrophic marine Nitrososphaerota (HMN) MAGs, which were subsequently combined with publicly available genomes to compile a final dataset of 104 HMN genomes (Table S2).

#### Global distribution of HMN

One hundred and four HMN genomes were used to recruit reads from 272 metagenomic and 156 metatranscriptomic libraries, encompassing data from this study as well as from the *Tara Oceans* and *Malaspina* expeditions (Tables S1 and S3). Metagenomic and metatranscriptomic raw reads were quality-controlled using fastp v0.20.0 [20], and rRNA sequences were removed from metatranscriptomic datasets using SortMeRNA v4.3.6 [25]. Read mapping was performed using Bowtie2 v2.4.1 [26], and relative abundance was quantified as RPKM (reads per kilobase per million mapped reads).

#### Comparison of basic genomic characteristics

Comparative genomic analyses were performed on HMN alongside representative marine prokaryotic lineages, including marine ammonia-oxidizing archaea, SAR11, SAR86, *Prochlorococcus*, OM43, and 2194 *Tara Oceans* MAGs (Table S2). Genomic features—such as GC content, gene count, coding density, and genome size were calculated using CheckM v1.1.3 [23]. The average number of nitrogen and carbon atoms per amino acid residue side chain, and the relative abundance of individual amino acids—were calculated as described by Mende et al. [27]. Orthologous gene clusters (OGs) were subsequently identified from all analyzed genomes using OrthoFinder v2.5.4 [28] for pan-genomic analysis.

#### Comparative metabolic analysis among genomes

Functional metabolism was compared among HMN, MAOA, and other typical heterotrophic archaea (non-Nitrososphaerota), including genomes affiliated with Bathyarchaeota and Euryarchaeota (Table S2). Gene annotation was performed using the Kyoto Encyclopedia of Genes and Genomes (KEGG) database [29], the archaeal clusters of orthologous genes database [30], the dbCAN database [31], and the MEROPS database [32], supplemented by the Rapid Annotation using Subsystems Technology server. Metabolic reconstructions accounted for genome completeness and contamination to infer gene presence statistically. HMN transcriptional activity was quantified from metatranscriptomic data, summarized at the KEGG orthology level, and further analyzed across different ecotypes.

#### Probe design and catalyzed reporter deposition fluorescence in situ hybridization

HMN- and MAOA-specific probes targeting 16S rRNA and *amoA* genes, respectively, were designed based on conserved regions identified from alignments of representative genomes (Supplementary Methods, Figs. S1 and S2). In situ seawater samples collected from multiple depths at seven stations in the Western Pacific Ocean were fixed, filtered, and subjected to a two-round CARD-FISH protocol in which MAOA- and HMN-specific probes were sequentially hybridized. Hybridized samples were visualized by confocal microscopy, and probe performance was validated against pure cultures of *Nitrosopumilus maritimus* SCM1 and negative control experiments with the nonspecific probe NON338.

#### Phylogenomic tree construction, molecular dating, and gene gain and loss

A phylogenomic tree was constructed using 53 archaeal marker genes [24] under the maximum-likelihood criterion in IQ-TREE v2.2.2.3 [33]. Utilizing these 53 optimized marker genes minimizes the influence of horizontal gene transfer and improves the recovery of the monophyletic lineages [34]. Divergence times were estimated with MCMCTree in PAML v4.9 [35], employing seven calibration nodes. Two independent MCMCTree runs were performed to confirm convergence of results (Fig. S3). Gene gain and loss events were reconstructed from orthogroups inferred by OrthoFinder v2.5.4 [28] using Dollo parsimony as implemented in COUNT [36].

#### Potential gene exchange between HMN and other microorganisms

Horizontal gene transfer (HGT) between HMN and other microorganisms was assessed using BLASTP-based similarity searches implemented in Diamond [37]. Phylogenetic relatedness was further evaluated with 31 conserved orthologous proteins [38] (Table S4) identified by eggNOG-mapper v2.1.12 [39]. Genes showing high sequence similarity between phylogenetically distant genomes were considered candidate HGT events [40]. Additionally, HGT-derived genes were identified using HGTector v2.0 [41], which performed Diamond BLASTP searches against the NCBI-nr protein database.

## Results and discussion

### Co-occurrence of HMN with marine AOA

We investigated the horizontal and vertical distribution of 104 HMN genomes (103 metagenome-assembled genomes, MAGs, and one single-cell amplified genome, SAG) on a global scale by mapping them to 272 metagenomes and 156 metatranscriptomes from global oceans, covering multiple size fractions (from <0.22 to >10 μm) and depths, including epipelagic (0–200 m), mesopelagic (200–1000 m), and bathypelagic (>1000 m) zones (Tables S1 and S3). HMN were predominantly found in the picoplankton size fraction of 0.22–3 μm which included free-living prokaryotes (Fig. S4), suggesting that the majority of HMN either had a free-living lifestyle or were associated with nano- or picoplankton. The overall relative abundances of HMN in metagenomes and metatranscriptomes from the bathypelagic (BAT) and mesopelagic (MES) zones were significantly higher than those from the epipelagic zones (Mann–Whitney test, *P* < 0.01; Fig. S4).

**Figure 1:**
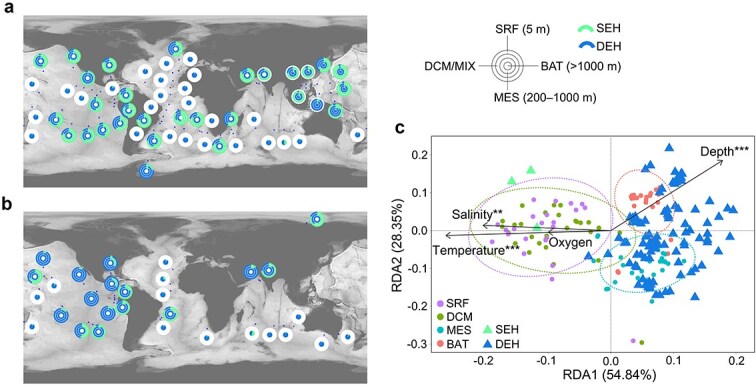
Global distribution of heterotrophic marine Nitrososphaerota (HMN). (a) The proportions of the shallow ecotype (SEH) and deep ecotype of HMN (DEH) among the total 104 HMN genomes, based on read recruitment from metagenomes and (b) metatranscriptomes across regions and depths. White color indicates no sample. (c) Redundancy analysis of 148 metagenomic samples based on RPKM profiles of 104 HMN genomes under environmental parameter constraints. ^**^P < 0.01, ^***^P < 0.001 (Monte Carlo permutation test). SRF, surface water; DCM, deep chlorophyll maximum layer; MIX, mixed layer; MES, mesopelagic zone; BAT, bathypelagic zone.

Nearly all HMN genomes exhibit their highest relative abundance in the BAT/MES zones, with the exceptions of MAG1, MAG2, and UBA539, which show higher relative abundance in the epipelagic zone (Mann–Whitney test, *P* < 0.05–0.01; Fig. S5A). A similar distribution pattern was observed in the metatranscriptomic dataset (Fig. S5B). It should be noted that relative abundance profiles do not directly reflect absolute abundance. Nevertheless, previous qPCR studies have consistently demonstrated higher absolute abundances of total HMN in the aphotic zone [13, 17]. Furthermore, among HMN natural populations, the lineages represented by MAG1, MAG2, and UBA539 are predominant in the global epipelagic zone, while the lineages represented by the other genomes account for a higher overall proportion in the MES/BAT (Figs. 1a and 1b). Based on these findings, we can classify the lineages represented by MAG1, MAG2, and UBA539 as the shallow ecotype of HMN (SEH), and the lineages represented by the remaining genomes as the deep ecotype of HMN (DEH). Redundancy analysis (RDA) of global metagenomes, using the HMN genomes’ abundance matrix and the environmental dataset as response and explanatory matrices, respectively, demonstrated that water mass characteristics (temperature, salinity, and depth) may be explanatory factors in the formation of the two ecotypes of HMN (Fig. 1c). Specifically, SEH were positively correlated with temperature and salinity but negatively correlated with depth, whereas DEH showed the opposite pattern, being positively correlated with depth and negatively correlated with temperature and salinity. Indeed, genomic comparisons further revealed that SEH exhibits potential adaptations to shallow water conditions, including high ultraviolet (UV) radiation, elevated reactive oxygen species (ROS) levels, and low nutrient concentrations, whereas DEH shows enhanced resistance to deep-sea environmental stressors, particularly cold temperatures (details in supplementary information).

**Figure 2:**
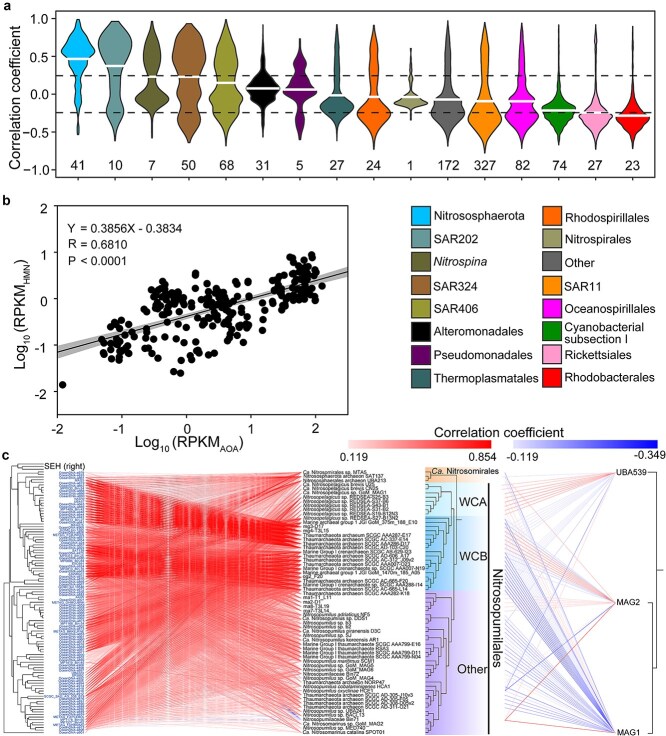
Co-occurrence of heterotrophic marine Nitrososphaerota (HMN) with marine ammonia-oxidizing archaea (MAOA). (a) Correlation coefficients between the relative abundances of 104 HMN genomes and the top 1000 most abundant marine prokaryotic operational taxonomic units (OTUs) based on 16S rRNA gene. Correlations falling between the black dashed lines were not statistically significant (Pearson correlation test *P* > 0.05). The solid white lines in the violin plots indicate the mean values. Numbers shown at the bottom represent the number of OTUs in each group. (b) Plot of the log-transformed sums of reads per kilobase of genome length per million of total metagenome reads (RPKMs) for 63 MAOA genomes versus the log-transformed sums of RPKMs for 104 HMN genomes. The line and shadow represent the linear regression line and the 95% confidence interval, respectively. (c) Correlation coefficients between the relative abundances of 104 HMN genomes and 63 marine AOA genomes. Only correlation coefficients with *P* < 0.05 (Pearson correlation test) are displayed. Average linkage hierarchical clustering of HMN genomes based on Bray–Curtis dissimilarity distances of range-standardized RPKMs is shown on the left (deep ecotype of HMN) and right (shallow ecotype of HMN, SEH). The phylogenomic tree of 63 marine AOA genomes is shown on the middle. WCA, water column group A; WCB, water column group B.

To explore potential associations, we correlated the relative abundances of 104 HMN genomes with the top 1000 most abundant prokaryotic 16S rRNA gene operational taxonomic units (OTUs, from the *Tara Oceans* metagenomics dataset [42]) (Fig. 2). The results revealed that HMN had the highest average positive correlation coefficient with Nitrososphaerota, primarily composed of AOA, although the overall strength of this correlation was moderate (Pearson correlation test, average *r* = 0.47; 84.0% of correlations significant at *P* < 0.05; Fig. 2a). Using competitive fragment recruitment analysis of 272 global metagenomes, we also found the total relative abundance of natural populations recruited to the 104 HMN genomes correlated positively with those recruited to the 63 representative MAOA genomes (Pearson correlation test, *r* = 0.68*, P* < 0.01) (Fig. 2b). Among 101 DEH genomes, 64.4% exhibited the strongest positive correlations with members of the deep marine AOA clade (water column group B, WCB), 24.8% showed the highest correlations with relatively shallow water column group A (WCA), and ~ 3% showed the highest correlations with the AOA genus *Nitrosopumilus*. In contrast, for all three SEH genomes, 68.9% of their correlations pairs with MAOA members were negative (predominantly with WCB), whereas all correlations pairs with WCA members were exclusively positive. Additionally, correlations between SEH and *Nitrosopumilus* were mostly negative (24.2%), with only a small fraction (3.6%) being positive (Fig. 2c). These results demonstrate a close spatial association between HMN and MAOA, consistent with their shared vertical distribution pattern: negligible surface abundance, peak at ~200 m, and gradual decrease with depth [13]. Notably, this association appears to be ecotype-specific, with SEH preferentially associating with WCA and DEH with WCB. It is generally believed that AOA primarily occur at the bottom of the euphotic zone to avoid light inhibition and competition from phytoplankton for fixed nitrogen [43, 44]. However, the metabolic strategies underlying HMN’s niche convergence with MAOA in the deep sea require further genomic investigation.

### Shared and distinct genomic traits in HMN versus marine AOA

**Figure 3:**
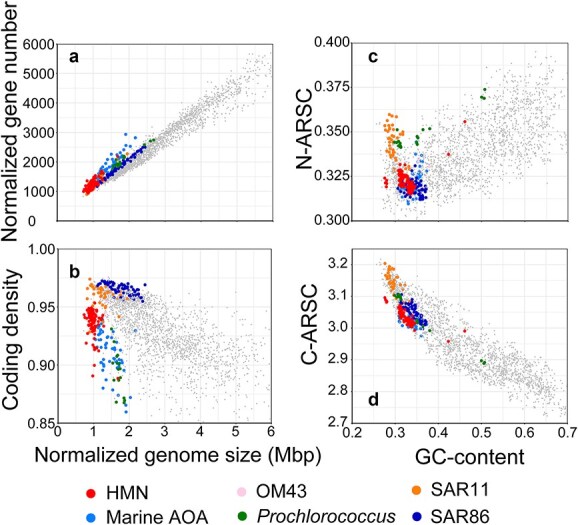
Comparison of basic genomic characteristics between heterotrophic marine Nitrososphaerota (HMN) and other typical marine microbes. (a) Completeness-normalized gene count and (b) coding density versus completeness-normalized genome size. (c) Average nitrogen (N-ARSC) and (d) carbon atoms per amino-acid-residue side chain (C-ARSC) versus genomic GC-content. Analyses include 104 HMN genomes, five model highly streamlined marine microbes, and 2107 *Tara Oceans* metagenome-assembled genomes (MAGs; gray dots). Only the data points within the displayed axes ranges are shown. AOA, ammonia-oxidizing archaea.

The 104 HMN genomes were compared with a total of 2310 genomes from free-living marine bacteria and archaea, including 2194 MAGs recovered from the *Tara Oceans* metagenomic datasets and 116 MAGs/strain genomes obtained from the literature. The comparative analysis specifically centered on HMN and well-known highly streamlined genomes of cosmopolitan marine microbial groups such as MAOA, alphaproteobacterial Pelagibacterales SAR11, gammaproteobacterial SAR86, betaproteobacterial OM43, and cyanobacterial *Prochlorococcus* (Fig. 3, Fig. S6, Fig. S7) [45–49]. The HMN genomes are distinguished by their exceptionally small genome size (ranging from 0.73 to 1.68 Mbp, with an average of 0.97 Mbp) and gene count (from 938 to 2185, averaging 1212) among these genomes (Fig. 3a, Table S2). Such an ultra-streamlined genome size of HMN is smaller than that of the previously known most streamlined free-living marine bacteria, SAR86 [49], and comparable to those of known symbiotic microbes such as Candidate Phyla Radiation bacteria and DPANN archaea, which typically range from 0.5–1.5 Mbp [50]. Moreover, HMN genomes exhibit gene coding densities (88.9%–95.3%) comparable to DPANN archaea (~85%–95%) [51], but slightly lower than SAR11 and SAR86 bacteria (Fig. 3b). This reduced gene coding density relative to typical streamlined free-living bacterial groups appears to be a conserved feature of streamlined archaeal genomes, likely reflecting their longer noncoding DNA regions associated with regulatory elements [52].

HMN genomes show low GC content (27.6%–46.1%; mean 32.6%), as well as low nitrogen content (Average number of nitrogen atoms per amino-acid-residue side chain, N-ARSC: 0.315–0.356) and high carbon content in encoded amino acid residue side chains (Average number of carbon atoms per amino-acid-residue side chain, C-ARSC: 2.958–3.096), highly similar to those of MAOA (30.9%–37.3%, 0.310–0.344, and 2.975–3.073; Figs. 3c and 3d). This, together with their significantly positive correlation in oceanic abundance patterns (Fig. 2), indicates that HMN and MAOA might share evolutionary adaptations to niches with similar nutritional conditions. Based on the ratio of pan-genome genes to core-genes [53], HMN showed a degree of pan-genome openness similar to MAOA, which fell between proteobacterial SAR11/SAR86 and cyanobacterial *Prochlorococcus* (Fig. S6). This finding further demonstrates that HMN and MAOA—these two phylogenetically related Nitrososphaerota groups—have evolved convergent genomic features, likely driven by niche similarity and shared environmental selection pressures [54], even as they employ contrasting trophic strategies (heterotrophy versus autotrophy).

Notably, while HMN and MAOA share similar N-ARSC and C-ARSC, their genomes encode significantly different relative abundances of individual amino acids, pointing to distinct metabolic fine-tuning within shared environmental constraints. (Fig. S7). For example, asparagine, isoleucine, and serine were much more abundant, while cysteine, glutamic acid, histidine, methionine, and valine were much more deficient in HMN than in MAOA (*P* < 0.01, Mann–Whitney test). The lower relative abundance of sulfur-containing cysteine and methionine in the encoded proteins of HMN compared with MAOA may result from deficiencies in pathways related to sulfur metabolism (as indicated by our metabolic analysis below). In addition, the lower efficiency of the cobalamin-independent methionine synthesis pathway in HMN, compared to the cobalamin-dependent pathway in MAOA [10], may further contribute to the reduced availability of methionine in HMN. The significantly higher relative abundance of serine in HMN may be a compensation for the lower relative abundance of cysteine, given their similar steric and chemical properties [55]. Notably, the extremely high serine content in encoded proteins was reported as a consistent characteristic of prokaryotic parasites (eg, Chlamydiae) with small genomes [56]. Taken together, these results indicate potential differences in genomic features and metabolic functions of HMN and MAOA, despite their shared ecological niches.

### Metabolic reliance of HMN on marine AOA

**Figure 4:**
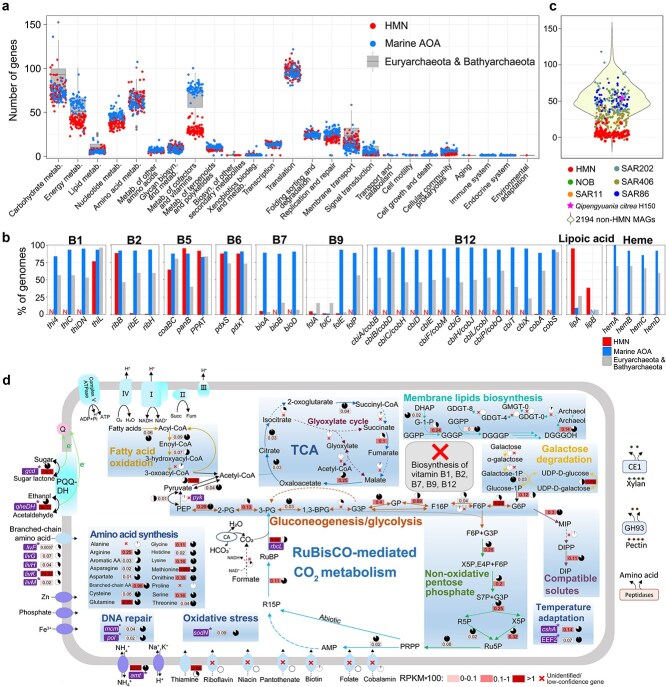
Metabolic characteristics of heterotrophic marine Nitrososphaerota (HMN). (a) Comparison of non-redundant functional gene counts (normalized by genome completeness) among HMN, marine ammonia-oxidizing archaea (MAOA), as well as Bathyarchaeota and Euryarchaeota across 26 KEGG categories. (b) Comparative analysis of vitamin and cofactor biosynthesis gene prevalence in the same four archaeal groups. (c) Comparison of vitamin and cofactor metabolic gene counts (non-redundant, genome-completeness-normalized) between HMN and 2195 non-HMN genomes (including 2194 *Tara Oceans* metagenome-assembled genomes (MAGs) and the *Qipengyuania citrea* H150 reference genome). (d) Metabolic reconstruction overview of HMN. Solid arrows represent confidently annotated genes, while dashed arrows and crosses (x) denote either unidentified genes or genes with extremely low prevalence probability below estimated cutoff (defined in methods). The transcriptional levels of key genes are presented as RPKM values (see methods). The accompanying pie chart displays the detection frequency of each gene across all analyzed HMN genomes. Key genes discussed in the main text are labeled with gene abbreviations (explained in Table S6). Genes involved in horizontal gene transfer are marked with asterisks (*).

In comparison to the representative genomes of MAOA and other dominant/typical heterotrophic archaeal groups, such as Bathyarchaeota and Euryarchaeota (Table S2), HMN exhibited a significant deficiency in genes involved in energy metabolism and the metabolism of cofactors and vitamins (*P* < 0.01, Mann–Whitney test) (Fig. 4a, Table S5). Unlike MAOA, Bathyarchaeota, or Euryarchaeota, which encode diverse pathways for carbon fixation, nitrogen metabolism, and/or sulfur metabolism (as indicated by the presence of representative enzymes such as 3-hydroxypropionyl-coenzyme A dehydratase, acetyl-CoA/propionyl-CoA carboxylase, nitrite reductase, ammonia monooxygenase, sulfite reductase, and sulfonate transport system), HMN lack these core energy metabolisms (Table S5). Genome-based analysis of metabolic potential demonstrated that HMN are obligate chemoorganoheterotrophs using oxygen as the sole electron acceptor, as reported in previous studies [10, 16]. Notably, HMN lack complete pathways for the synthesis of vitamin B_1_ (thiamine), B_2_ (riboflavin), B_7_ (biotin), B_9_ (folate), and B_12_ (cobalamin), in sharp contrast to MAOA, which possess all of these pathways (Fig. 4b, Table S5). Beyond known symbiotic or parasitic microbes, HMN could constitute the marine microorganism with the most streamlined vitamin metabolism identified to date (Fig. 4c). The absence of multiple vitamin synthesis pathways has also been reported in other non-AOA (basal) subclades of Nitrososphaerota, which primarily inhabit vitamin-rich terrestrial and hydrothermal environments [57–59]. A well-studied example of vitamin auxotrophy is *Pelagibacter ubique*, a streamlined SAR11 bacterium that lacks biosynthetic pathways for vitamins B_1_, B_5_, B_6_, B_7_, and B_12_, and may rely on microbes such as *Prochlorococcus* for nutrient acquisition [60, 61]. Similarly, HMN may fulfill its B-vitamin requirements through interactions with dominant vitamin producers like MAOA, which are both prevalent in deep-sea environments and possess complete biosynthetic pathways for most B vitamins (particularly vitamin B_12_) (Fig. 4b). This is supported by the prevalence of AOA-affiliated cobalamin synthesis genes in the depths of the global ocean [62], high cobalamin content per carbon basis in cultured AOA [63], and significant release of B vitamins by AOA based on culture experiments [64]. The auxotrophy of HMN for B vitamins may underlie its co-occurrence with MAOA in the deep sea. Indeed, the depth distribution of B vitamins was surprisingly consistent with that of HMN and MAOA, exhibiting peaks in the upper mesopelagic zone [65]. A previous co-culture study revealed B vitamin-dependent metabolic interactions between MAOA and the heterotrophic alphaproteobacterium *Qipengyuania citrea* H150, originally isolated from a nitrifier enrichment [66]. Compared with the cocultured *Q. citrea* H150 and ubiquitous marine free-living prokaryotes (eg, SAR11, SAR86, SAR202, SAR406, and nitrite-oxidizing bacteria), HMN possess the most streamlined vitamin/cofactor biosynthesis pathways, making them a potential symbiotic partner for AOA in the natural marine environment (Fig. 4c).

Furthermore, deep-sea-dwelling HMN were found to express abundant, if not the most abundant, PQQ-dependent alcohol dehydrogenase (ADH) for energy acquisition (see details below), yet they completely lack the heme C biosynthesis genes (*hemABCD*), which are typically required as prosthetic group for ADH (Fig. 4b). Complementarily, MAOA contain all of these genes (Fig. 4b). In contrast to MAOA and other heterotrophic archaea, only HMN contain the complete set of genes (*lipA* and *lipB*) for lipoic acid synthesis, an important coenzyme for oxygen-tolerant pyruvate dehydrogenase [67] (Fig. 4b). Lipoic acid is also well known as an antioxidant involved in the detoxification of reactive oxygen species (ROS) [68, 69], which has been reported to largely inhibit the activity of AOA [70–73]. In this context, the lipoic acid released from HMN cells could detoxify ROS for AOA. In addition, HMN possess all key enzymes required for archaeol synthesis (Fig. 4d). However, unlike AOA, they typically lack tetraether synthase (Tes) for converting archaeol into glycerol dialkyl glycerol tetraethers (GDGTs)—lipids that enhance membrane stability under extreme conditions. The co-occurance between HMN and MAOA may influence the Tex_86_ paleotemperature proxy via their combined effects, particularly in co-dominated deep-sea systems [74], which warrants future research.

In terms of organic carbon metabolism, the most actively expressed genes in metatranscriptomes with high occurrence rates among the 104 HMN genomes were associated with PQQ-dependent dehydrogenases (*qheDH* and *gcd*), branched-chain amino acid (BCAA) transport system protein (*livK*), and RuBisCO (*rbcL*) (Fig. 4d). This is consistent with previous findings on HMN’s metabolism, which indicate that HMN utilize membrane-bound PQQ-dependent dehydrogenases to oxidize various extracellular carbon compounds, directly channeling reducing equivalents into the electron transport chain for energy acquisition. In addition, HMN engage in BCAA-related fatty acid metabolism and RuBisCO-mediated CO_2_ incorporation, supporting potential biosynthesis in energy-limited deep water [10, 16]. Notably, genes encoding succinyl-CoA synthetase and fumarate hydratase—key citrate cycle enzymes responsible for the conversion of succinyl-CoA to succinate and fumarate to malate, respectively—as well as those for the glyoxylate cycle, were absent in nearly all HMN genomes (Fig. 4d). This implies that HMN hardly perform the complete citrate cycle. In addition, the *pyk* gene, which encodes pyruvate kinase related to glycolysis, was not found in HMN (Fig. 4d). These results suggest that HMN may depend on exogenous substrates and intermediates for essential metabolic activities.

The high expression of amino acid transporter Liv in HMN suggests active uptake of exogenous amino acids (Fig. 4d). This strategy may provide an energy-efficient nitrogen and carbon source in the energy-limited deep sea, as transport requires less energy than de novo synthesis. Amino acid transporters in deep-sea dwelling HMN may possess a high affinity for their substrates, as confirmed by the active uptake of amino acids by deep-sea archaea at natural concentration (nanomolar) through autoradiography [75]. Moreover, deep-sea archaea are believed to efficiently utilize D-amino acids, an important constituent of the marine recalcitrant dissolved organic matter (RDOM) pool [76]—a capacity not shared by most bacteria [77]. Thus, the HMN-MAOA co-occurrence may result in sharing of ammonia, granting access to the RDOM pool through HMN-mediated regeneration and circumventing the limited remineralization efficiency of heterotrophic bacteria. Consistent with this, ammonium transporters (Amt) are highly expressed in HMN (Fig. 4d). While Amt transporters are generally associated with ammonium uptake, a previous study on bacteria suggested that they may also facilitate the efflux of ammonia from the cell [78]. In turn, MAOA may represent a potential source of organic carbon and nitrogen for HMN, as experimentally demonstrated by their release of various amino acids [64].

### Cellular association of HMN with marine AOA

**Figure 5:**
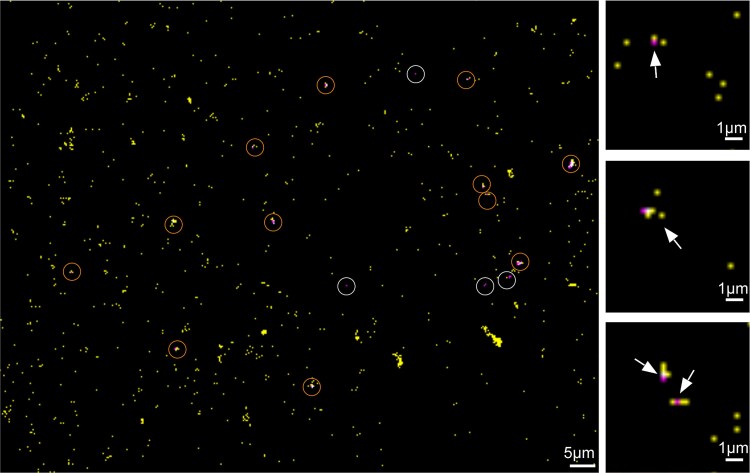
Cellular association of heterotrophic marine Nitrososphaerota (HMN) with marine ammonia-oxidizing archaea (MAOA). Catalyzed reporter deposition fluorescence in situ hybridization image showing HMN (magenta) and MAOA (yellow) detected with specific fluorescent probes. Orange circles mark HMN cells showing cellular association with MAOA; white circles mark free HMN cells. Due to the use of oligonucleotide probes (targeting 16S rRNA or *amoA* genes), cells in CARD-FISH images appear as bright spots rather than complete outlines, which is characteristic of this method.

While metabolic analysis indicates that HMN lacks the capacity for de novo vitamin biosynthesis, it is noteworthy that genes encoding corresponding vitamin transporters are also absent in nearly all HMN genomes (Table S7). This suggests that their acquisition of vitamins may occur through non-transporter-mediated pathway, potentially facilitated by direct physical contact with host cells. To test this hypothesis, we performed CARD-FISH using MAOA- and HMN-specific probes. CARD-FISH imaging revealed that HMN cells constituted approximately 2.1% of total MAOA cell counts in situ (Figs. 5, S8, and  S9), which aligns with previous estimates (mean: 1.4%) based on HMN-specific RuBisCO and thaumarchaeal *amoA* gene abundances [10]. Negative control experiments using the nonspecific probe NON338 showed no detectable false positive signals (Figs. S10 and S11). Notably, approximately 83.8% of HMN cells exhibited direct physical contact with MAOA cells, from our count of 98 HMN cells across six fields of view (Figs. 5 and S9). Given that fluorescent labeling failed to detect ~27.6% of MAOA cells (Fig. S12), we speculate that the remaining 16.2% of HMN cells could associate with those unlabeled partners. This finding, to our knowledge, represents the first documented evidence of an intimate archaea-archaea association in pelagic marine environments. In contrast, the DPANN archaea—which are known to be involved in inter-archaeal symbiosis—are typically documented in extreme habitats such as hydrothermal vents, hypersaline lakes, and deep-sea sediments [79]. The observed cell–cell contacts may facilitate close-range metabolic exchange between HMN and MAOA. Such interactions are reminiscent of symbiotic systems that bypass canonical membrane transport via direct cytoplasmic connections—a mechanism analogous to the *Ignicoccus hospitalis*-*Nanoarchaeum equitans* symbiosis [80, 81]. However, direct evidence for these specific exchange mechanisms in the HMN-MAOA association remains to be established.

### Co-origin of HMN with AOA

**Figure 6:**
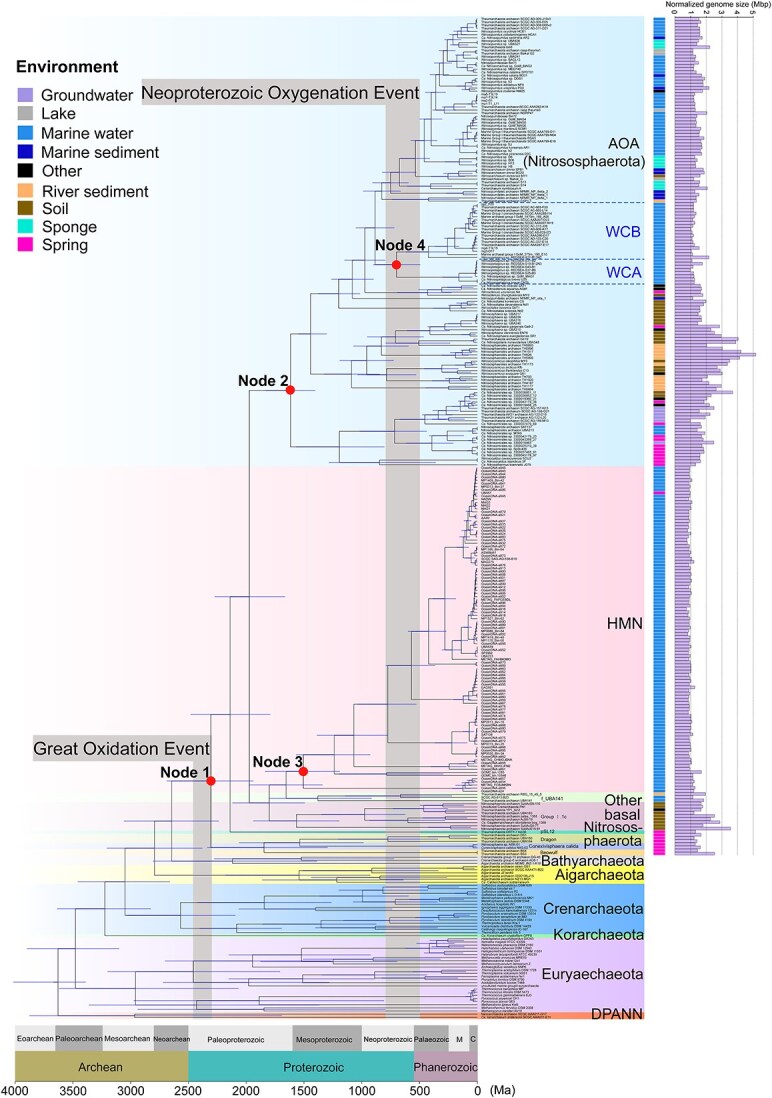
Maximum-likelihood phylogenomic tree of 104 heterotrophic marine Nitrososphaerota (HMN) genomes, 163 non-HMN Nitrososphaerota genomes, and 52 non-Nitrososphaerota archaeal genomes, with evolutionary timeline constrained by an old archaeal root age. Two vertical gray columns represent the Great Oxidation Event (~2.46–2.3Ga) and the Neoproterozoic Oxygenation Event (~0.8–0.5Ga) separately. Horizontal blue bars represent the 95% highest posterior density confidence interval. The sources of genomes are indicated by colored squares. Nodes 1 to 4 indicate the origin times of Nitrososphaerota, ammonia-oxidizing archaea (AOA), the HMN, and the WCA (water column group A) and WCB (water column group B) clades of AOA, respectively. M, Mesozoic; C, Cenozoic. Column charts display the sizes of genomes normalized by their completeness.

The timeline of HMN’s evolution and its evolutionary relationship with AOA were further explored using the classical model MCMCTree [35] (Fig. 6). Nitrososphaerota originated around 2305 Ma (node 1; 95% cofidence interval [CI]: 2687–1937 Ma), associated with the Great Oxidation Event (GOE; the first significant buildup of atmospheric oxygen, ~2.46–2.3 Ga). Following this, AOA (node 2: 1617 Ma; CI 1835–1402 Ma) and HMN (node 3: 1505 Ma; CI 1837–1189 Ma) emerged in close succession from the terminal Paleoproterozoic to the incipient Mesoproterozoic (Fig. 6). Our results are consistent with previous reports suggesting that AOA first evolved in terrestrial geothermal environments before being transferred to mesophilic soils and subsequently diversifying into marine ecosystems [57, 82] (Fig. 6). Notably, heterotrophic Nitrososphaerota exhibit a similar evolutionary trajectory: originating in hydrothermal systems (represented by thermophilic lineages such as Beowulf, Conexivisphaera, Dragon, and pSL12 at the root of the Nitrososphaerota branch), colonizing terrestrial habitats (represented by the aerobic Nitrososphaerota family group I.1c and clade UBA141), and finally radiating into the ocean (represented by the HMN group) (Fig. 6). However, a paradox arises from the observation that the origin of aerobic HMN predates the Neoproterozoic Oxygenation Event (NOE; ~0.8–0.5 Ga; Fig. 6), a period when oxygen levels became sufficient to ventilate the deep ocean [83]. This temporal discrepancy suggests that the earliest HMN members might not have relied on strict aerobic metabolism. Alternatively, they may have inhabited non-sulfidic, slightly oxygenated surface environments, where oxygen was locally available prior to the full ventilation of the deep ocean. In addition, they could have utilized alternative electron acceptors like nitrate or sulfate, as observed in some non-HMN basal Nitrososphaerota [1, 84], or inhabited non-marine environments (eg, terrestrial soil or geothermal systems), where oxygen was more easily available from the atmosphere—similar to early members of AOA (Fig. 6). Those “non-marine” HMN members might have been missed in this study due to preferential collection of marine samples.

The typical marine clades of AOA (WCA and WCB) formed during the NOE (node 4: 699 Ma; CI 900–493 Ma). Following this divergence, HMN underwent a period of accelerated evolution, as evidenced by densely clustered, shallow branches at the terminus of the HMN clade (Fig. 6). This rapid evolution likely drove their metabolic adaptations toward obligate aerobiosis. Furthermore, the presence of highly dispersed, shallow subclades suggests the existence of multiple HMN microhabitats with ultra-small effective population sizes, as expected under conditions of intense genetic drift. These patterns, together with genomic streamlining, are consistent with features commonly observed in organisms exhibiting symbiotic associations [85, 86]. We therefore hypothesize two possible pathways for the evolution of HMN: (1) HMN initially thrived as free-living organisms, like basal Nitrososphaerota lineages with larger-genomes, in the sulfidic, oxygen-deficient Mesoproterozoic ocean [87], but later established close ecological associations with MAOA after the NOE enabled oxic conditions in the deep ocean. (2) HMN have maintained a persistent ecological association with AOA since their origin. They initially inhabited terrestrial environments, then entered the ocean alongside AOA following the NOE.

### Complementary evolution of HMN and AOA

**Figure 7:**
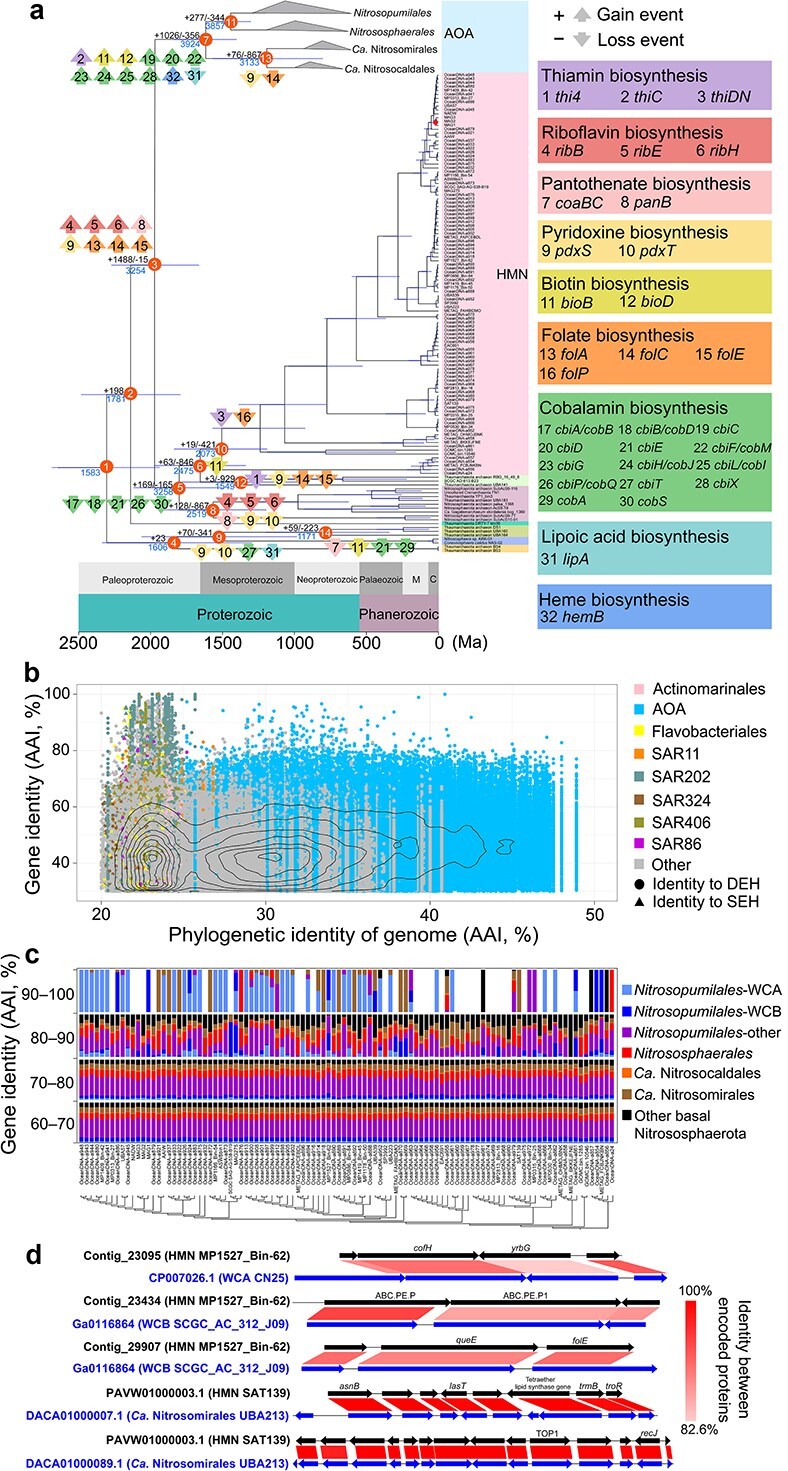
Analyses of gene gain/loss and potential horizontal gene transfer in heterotrophic marine Nitrososphaerota (HMN). (a) Dated phylogenomic tree indicating gene gain and loss events during the evolution of Nitrososphaerota. The three numbers near nodes 1 to 14 (in orange) indicate the counts (in black) of gained (+) and lost (−) orthologous gene clusters (OGs), as well as the total number (in blue) of OGs within the clades. The gain and loss events for the genes involved in vitamin and cofactor biosynthesis are marked with upward and downward arrows, respectively. The numbers on the arrows correspond to the gene abbreviations (explained in Table S6) on the right. (b) Comparison of gene-level and genome-level (based on 31 conserved orthologous genes) amino acid identities (AAIs) between *Tara Oceans* metagenome-assembled genomes (MAGs) and HMN genomes. The contour map displays the density distribution of data points. (c) Taxonomic composition of non-HMN Nitrososphaerota gene showing >60% AAI to HMN genes. The x-axis order of 104 HMN genomes reflects their phylogenomic relationships inferred from 53 archaeal marker genes (see tree below). (d) Selected long genomic fragments from HMN (in black) and marine ammonia-oxidizing archaea (MAOA, blue) encoding multiple high-identify proteins (abbreviations explained in Table S6). AAIs between proteins are represented by red-gradient parallelograms. Direction of the arrow indicates transcription direction of the gene. Names of contigs containing these genomic fragments are shown on the left. SEH, shallow ecotype HMN; DEH, deep ecotype HMN; WCA, water column group A; WCB, water column group B.

The analysis of gene gain and loss events indicated that gene losses vastly outnumbered gains (421 vs. 19) on the branch leading to HMN (node 10) but that gains far exceeded losses (1026 vs. 356) on the branch leading to AOA (node 7; Fig. 7a), a pattern consistent with a previous study [10]. Numerous genes involved in the biosynthesis of vitamins and cofactors were lost during the transformation from the common ancestor (node 3) of HMN and AOA to HMN, while many additional vitamin-biosynthesis related genes were gained during the transition from node 3 to AOA (Fig. 7a). This suggests opposite evolutionary strategies for HMN and AOA in the acquisition of vitamins. The lost metabolic functions of the streamlined HMN may be compensated by co-occurring AOA that possess these functions, aiming to reduce the costs of cell maintenance and division in the energy-limited deep ocean, as proposed by the Black Queen Hypothesis [88].

In addition to massive gene loss, potential gene acquisition through HGTs—a primary driving force behind evolutionary innovation in archaea [89, 90]—may also contribute to the evolutionary trajectory of HMN diverging from MAOA. Protein sequences from 104 HMN genomes were blasted against those of 2304 non-HMN MAGs retrieved from the *Tara Oceans* metagenomics dataset to identify potential HGT events between HMN and other marine microorganisms. Surprisingly, several bacterial lineages—particularly SAR202 and SAR406, which are abundant in the deep ocean, as well as *Flavobacteriales*, prevalent in the upper ocean—exhibited numerous proteins with high identity (>80% amino acid identity, AAI) to those in deep and shallow ecotypes of HMN, respectively, despite their phylogenetic distance from HMN (Fig. 7b). This finding may reflect active HGT events between HMN and ambient bacteria. To investigate this, we applied the HGT detection tool HGTector to both HMN and AOA genomes against the NCBI nr database [41]. The analysis revealed that 7.6% of HMN genes were horizontally acquired, of which 54.8% originated from bacteria and 1% from archaea (Fig. S13A). In contrast, 7.9% of AOA genes were horizontally acquired, with 64.9% derived from archaea and 10.1% from bacteria. However, this tool is not effective in detecting HGTs between AOA and HMN due to their ambiguous phylogenetic relationship in the nr database. The genes transferred from bacteria may have contributed to the development of a heterotrophic lifestyle of HMN, consistent with the observation that a high proportion of these transferred genes are involved in carbohydrate metabolism (~13.43% of total HGTs from annotated bacteria), amino acid metabolism (8.66%), and unclassified metabolism (8.75%) (Fig. S13B). In particular, the highly expressed PQQ-dependent dehydrogenase and BCAA transport system protein genes (*qheDH* and *liv*), which may be crucial for HMN’s heterotrophic lifestyle, were likely acquired through HGT (Fig. 4d). In addition, genes related to temperature adaptation and oxidative damage resistance, such as *uvrD, sodN*, and *cshA*, are also associated with HGTs from bacteria (Fig. S14). Thus, HGTs from bacteria may have driven the expansion of HMN’s ancestor from hyperthermal and anoxic environments to cooler, oxygenated deep-sea habitats [91].

The close spatial association between HMN and MAOA may also contribute to the frequency of HGT events between them. The BLASTP alignments of protein sequences from 104 HMN genomes and 163 non-HMN Nitrososphaerota genomes (Table S2) revealed that the proteins with the highest identity (>90% AAI) to HMN predominantly originate from AOA affiliated with WCA, WCB, and *Ca.* Nitrosomirales (Fig. 7c), despite these clades not being HMN’s closest relatives according to 53 core gene phylogeny (Table S8). Among these proteins, the most prevalent (found in 41 of 104 HMN genomes) was LeuC, the large subunit of 3-isopropylmalate dehydratase involved in leucine biosynthesis, which shared >90% AAI with WCA-affiliated AOA homologs (Table S9). In addition, the HMN genomes MP1527 and SAT139 contain several long genomic fragments encoding multiple proteins that are highly similar (82.6–100%) to those in WCA-, WCB- and *Ca.* Nitrosomirales-affiliated genomes (Fig. 7d, Table S9). Most of the annotated proteins in these fragments are related to transcriptional regulation, DNA recombination and repair, and membrane transporters (Fig. 7d, Table S9). The frequent exchange of recombination and repair-related genes was also observed between crenarchaeon *I. hospitalis* and its symbiont *N. equitans* [92], which may enhance the tolerance of recipient organisms to DNA damage. Processing DNA damage may be especially crucial for cells with a streamlined genome, as any DNA damage can significantly impact cellular functions. Although the aforementioned gene exchanges may be biased due to potential contamination from other microbes in MAGs during binning, the top gene identical to that in the single-cell genome AG-538-B19 of HMN originates from SAGs of WCB (Table S9), supporting the existence of potential HGTs between HMN and MAOA. Taken together, these findings demonstrate a close coevolutionary relationship between HMN and MAOA.

## Conclusion

We integrate large-scale molecular analyses and CARD-FISH imaging to suggest that HMN and MAOA form a close ecological association marked by persistent cell-to-cell physical interactions and overlapping environmental distributions. Their shared genomic backbone contrasts with specialized amino acid composition and complementary metabolic traits—most notably, HMN acquired heterotrophic capabilities through bacterial gene transfer while losing vitamin biosynthesis pathways, a deficiency that might be compensated by MAOA’s retention and acquisition of these vitamin-related genes. This metabolic interdependence, potentially sustained by continuous HGT, reflects a coevolutionary alliance that originated after the GOE and has been maintained over extended evolutionary timescales. This study therefore reveals a previously unrecognized close association between HMN and MAOA, although whether this relationship is obligate or facultative remains to be elucidated. This lifestyle resolves the paradox of HMN’s exceptionally small genome size in deep-sea environments—a striking anomaly compared to other deep-sea planktonic prokaryotes, which typically evolve larger genomes than their epipelagic counterparts (possibly due to relaxed purifying selection under conditions of greater organic compound diversity and smaller population sizes in the deep sea) [93]. Future research should overcome challenges of cultivating HMN by establishing HMN-MAOA co-culture system, combined with single-cell metabolic flux analysis to validate these findings. Nevertheless, our findings provided evidence for a previously unrecognized association involving ubiquitous marine AOA clades. As the dominant chemolithoautotrophic primary producers in the deep ocean, MAOA were previously known to include only a few members that forming symbiotic associations, exclusively with marine invertebrates such as sponges [94–96]. Our discovery suggests a novel coupling between ammonia-oxidizing and heterotrophic Nitrososphaerota, parallel to the well-established ammonia oxidizer-nitrite oxidizer partnership in deep ocean ecosystems [97, 98]. Such a close partnership suggests an immediate interconnection between primary and secondary production, potentially driving much faster carbon turnover in the deep sea than previously recognized.

## Supplementary Material

Supplementary_material_ycag173

## Data Availability

All raw sequences and metagenome-assembled genomes produced from this study are deposited in the National Center for Biotechnology Information (NCBI) under BioProject IDs PRJNA1158446, PRJNA1163519, and PRJNA1089822. For the analyzed existing, publicly available datasets, their accession numbers are listed in Table S2 and Table S3. Any additional information required to reanalyze the data reported in this paper is available upon request.
