# Supplementary material for: Close spatial and metabolic association between heterotrophic and ammonia-oxidizing marine Nitrososphaerota
Source: ISME Commun. 2026 Jun 18;6(1):ycag173. doi: 10.1093/ismeco/ycag173 (PMC13374868; doi:10.1093/ismeco/ycag173)
Supplement: Supplementary_material_ycag173 [file supplementary_material_ycag173.zip › Supplementary information.docx]

**Supplementary information**

**Close spatial and metabolic association between heterotrophic and ammonia-oxidizing marine Nitrososphaerota**

Qian Li^1^, Mingming Chen^1, *^, Ye Lu^1^, Cheng Xu^1, 2^, Yue Zheng^3^, Zhirui Zeng^4^, Dapeng Xu^1^, Wei Qin^5^, Yao Zhang^1, 2, *^

^1^State Key Laboratory of Marine Environmental Science, College of Ocean and Earth Sciences, Xiamen University, Xiamen, Fujian 361102, China

^2^Advanced Institute for Marine Studies, Fujian Ocean Innovation Center, Xiamen, Fujian 361102, China

^3^College of the Environment and Ecology, Xiamen University, Xiamen, Fujian 361102, China

^4^Department of Ocean Science and Engineering, Southern University of Science and Technology, Shenzhen, Guangdong 518055, China

^5^Department of Microbiology, Carl R. Woese Institute for Genomic Biology, University of Illinois Urbana-Champaign, Urbana, IL, 61801, United States

*Corresponding author. Yao Zhang, State Key Laboratory of Marine Environmental Science, College of Ocean and Earth Sciences, Xiamen University, No. 4221 Xiangan South Road, Xiamen, Fujian 361102, China. Email: yaozhang@xmu.edu.cn (Y.Z.); Mingming Chen, State Key Laboratory of Marine Environmental Science, College of Ocean and Earth Sciences, Xiamen University, No. 4221 Xiangan South Road, Xiamen, Fujian 361102, China. Email: chenmingming@xmu.edu.cn.

**The PDF file includes:**

Supplementary methods

Supplementary results

References

Figures S1 to S14

**Supplementary methods**

**Sample collection and DNA extraction**

A total of 67 metagenomic samples were collected at 11 stations in the South China Sea (SCS) and the Western Pacific aboard the R/V *Tan Kan Kee* and *Ke Xue* during six cruises between 2019 and 2022. Temperature, salinity, dissolved oxygen concentration, and depth were measured using a conductivity–temperature–depth (CTD) system (SBE 9/17 plus; SeaBird Inc., USA). Approximately 20 to 570 liters of seawater were sequentially filtered through polycarbonate (Millipore) or glass fiber membranes (Advantec) with pore-sizes of 10 μm, 3/1.6/0.8 μm, and 0.3/0.2 μm (142 mm diameter) for DNA extraction. All membranes were flash-frozen in liquid nitrogen and then stored at -80℃ until further analysis. Detailed sampling information can be found in Table S1. DNA was extracted using the phenol-chloroform-isoamyl alcohol method as described by Massana et al. [1], and DNA concentrations were measured using a Qubit HS dsDNA Assay Kit (Invitrogen, Life Technologies).

**Metagenome sequencing, co-assembly, and binning**

Paired-end sequencing (2 × 150) was performed using the Illumina NovaSeq 6000 platform. Raw metagenomic reads for each sample were trimmed using fastp v0.20.0 [2]. The generated metagenomic data from the SCS samples were organized into different metagenomic sets based on their locations, depth zones, and size fractions, with each set undergoing individual co-assembly and binning. Co-assembly was performed using Megahit v1.2.9 [3] with a minimum scaffold length of 1 kbp. Then, the scaffolds were binned using Anvi’o v7 (https://merenlab.org/software/anvio) by integrating binning algorithms CONCOCT [4], MetaBAT2 [5], and MaxBin2 [6]. Generated metagenome-assembled genomes (MAGs) were dereplicated using dRep v3.4.2 [7] with a cutoff of 99% average nucleotide identity. The completeness and contamination of each MAG were evaluated using CheckM v1.1.3 [8]. These MAGs were classified using the Genome Taxonomy Database Toolkit (GTDB-Tk) [9]. Although taxonomic classification was performed using GTDB-Tk, taxonomic names reported in this study follow the NCBI taxonomy for consistency with existing literature. The MAGs closely related to the genome UBA57 were identified as heterotrophic marine Nitrososphaerota (HMN) [10]. The taxonomy of these candidate HMN MAGs was further confirmed by phylogenomic analysis of 53 concatenated archaeal marker genes (details in a later section). As a result, three MAGs (completeness > 79% and contamination < 5%) assigned to HMN (Table S2) were obtained for downstream analysis. Generated HMN-affiliated MAGs in this study, along with collected HMN-affiliated genomes from the literature and public databases, were further dereplicated using dRep v3.4.2 [7] with a cutoff of 99% average nucleotide identity. In total, we obtained 104 HMN genomes.

**Global distribution of HMN**

One hundred and four HMN genomes were used to recruit sequences from 272 environmental metagenomic libraries and 156 metatranscriptomics libraries, including 67 metagenomes obtained in this study, 153 published metagenomes and 141 published metatranscriptomes from the *Tara Oceans* expeditions, and 52 published metagenomes and 15 published metatranscriptomes from the *Malaspina* expeditions (Table S1 and S3). Quality control of metagenomic and metatranscriptomic reads was performed using fastp v0.20.0 [2]. For metatranscriptomic reads, rRNA sequences were removed using SortMeRNA v4.3.6 [11]. The high-quality reads were then competitively mapped to HMN genomes using Bowtie2 v2.4.1 [12] with default parameters. Genomic relative abundance was quantified using RPKM (reads per kilobase per million) with metagenomic reads normalized by genome length and metatranscriptomic reads by gene length (with per-genome summation of gene-normalized values). The proportion of each genome within the HMN community was calculated by dividing the recruited reads by that specific genome by the total recruited reads from all HMN genomes.

**Comparison of basic genomic characteristics**

One hundred and four HMN genomes, 63 marine ammonia-oxidizing archaea (MAOA) genomes, 13 SAR11 genomes, 21 SAR86 genomes, 16 *Prochlorococcus* genomes, and three OM43 genomes (obtained in this study and collected from published/publicly available databases; Table S2), along with 2,194 published MAGs recovered from the *Tara Oceans* metagenome datasets (with potential symbiont genomes manually removed based on GTDB-Tk [9] taxonomic annotation) [13], were used to compare the basic genomic characteristics. The completeness, contamination, GC content, coding density, and genome size of each genome were estimated using CheckM v1.1.3 [8]. Genes were predicted using Prodigal v2.6.3 [14] with default settings. The average number of nitrogen or carbon atoms per amino-acid-residue side chain (N-ARSC and C-ARSC) was calculated as described by Mende et al. [15]. Orthogroups (orthologous gene clusters, OGs) from all analyzed genomes (HMN, MAOA, SAR11, SAR86, *Prochlorococcus*, and OM43 genomes) were obtained using OrthoFinder v2.5.4 [16] with default parameters to perform pan-genomic analysis. All singletons were retained to prevent loss of genetic information. The average relative abundance of each amino acid in the encoded protein sequences of every genome was calculated by dividing the number of occurrences of each amino acid by the total length of the encoded proteins.

**Comparative metabolic analysis among genomes**

To compare functional metabolism among HMN, MAOA, and other typical heterotrophic archaea (non-Nitrososphaerota), 30 high-quality genomes (completeness > 95%, contamination < 5%) belonging to Bathyarchaeota and Euryarchaeota were obtained from published/publicly available databases (Table S2). Open reading frames (ORFs) for individual genomes were predicted using Prodigal v2.6.3 [14] with default settings. Genes were annotated using eggNOG-mapper v2.1.12 [17] with default parameters against the Kyoto Encyclopedia of Genes and Genomes (KEGG) database [18], a Diamond BLASTP search [19] against the archaeal clusters of orthologous genes database [20] (top hit with e-value < 10^−5^, identity > 35%, alignment length > 80, and bit score > 100), and the online Rapid Annotation using Subsystems Technology server (https://rast.nmpdr.org/). All annotations were manually checked by comparing the results from the three databases, and KEGG catalogs were used for subsequent analyses. Carbohydrate-active enzymes were identified using a hidden Markov model against the dbCAN database [21] (e-value < 10^−10^, coverage > 0.3). Peptidases were annotated using a Diamond BLASTP search [19] against the MEROPS database [22] with cutoffs of e-value < 10^−10^. Extracellular enzymes were predicted using SignalP6 v6.0 [23]. To compare functional genes involved in vitamin and cofactor metabolism between HMN and other ubiquitous or biogeochemically significant lineages, we also predicted the genes in 2,194 non-HMN MAGs from the *Tara Oceans* metagenomic dataset — after manually removing potential symbiont genomes based on GTDB-Tk [9] taxonomic annotation [13] — and a genome affiliated with the alphaproteobacterium *Qipengyuania citrea* H150 [24] using Prodigal v2.6.3 [14], followed by annotation with eggNOG-mapper v2.1.12 [17] using default parameters.

In metabolic reconstruction of either HMN, SEH, or DEH, the presence of a gene was determined using the following criterion: the probability that the gene derives exclusively from authentic sequences must exceed the probability of it originating solely from contaminants. This condition is formalized by the inequality (1):

*b*(*k*; *n*, *p_1_*) > *b*(*k*; *n*, *p_0_*) (1)

where *b()* denotes the binomial probability mass function, *k* is the observed gene frequency across genomes, *n* is the total number of genomes analyzed, *p_1_* represents the mean genome completeness, and *p_0_* represents the mean genome contamination.

The transcriptomic level of each HMN-associated gene was assessed by mapping quality-controlled non-rRNA metatranscriptomic reads to HMN genomes using Bowtie2 v2.4.1 [12] (Table S3). A RPKM value was calculated for each gene in each recruited metatranscriptome. The overall transcriptomic level of each gene was calculated as the mean RPKM values across all recruited metatranscriptomes. For each KEGG orthology (KO) group, HMN transcriptomic activity was calculated as the sum of average RPKM values of all HMN-associated genes, normalized by the total number of HMN genomes (n = 104). For ecotype-specific analysis, SEH and DEH genes were mapped to epipelagic and aphotic zone metatranscriptomes, respectively. Per-KO transcriptomic activity was calculated as: (Σ average RPKMs of SEH genes)/3 for SEH, and (Σ average RPKMs of DEH genes)/101 for DEH.

**Probe design and catalyzed reporter deposition fluorescence in situ hybridization**

An HMN-specific 16S rRNA probe (5’-TTAACTATGTRAGAAGTGTRG-3’) was designed to target a conserved region exclusive to HMN (absent in MAOA), based on multiple alignments of 16S rRNA gene sequences from 33 HMN and 44 MAOA representative genomes (Fig. S1). An MAOA-specific *amoA* gene probe (5’-CWCCATAYATGACBCCKATAG-3’) was designed to target a conserved region identified through alignments of *amoA* sequences from 6 WCA and 15 WCB representative genomes (Fig. S2). Multiple sequence alignments of 16S rRNA and amoA gene sequences were performed using MAFFT (v7.310) with default parameters [25]. The probe specificities were further verified by querying against the NCBI-nr and SILVA databases using BLAST (v2.12.0) or TestProbe 3.0.

In situ seawater samples for CARD-FISH analysis were collected from the Western Pacific Ocean aboard R/V Dongfanghong 3 during September‒October 2024. A total of 243 mL seawater from depths ranging from 100 m to 4000 m across seven stations in the Western Pacific Ocean (32.4°N‒42.2°N, 146.2°E‒159.3°E) was pooled to obtain an integrated sample representing a broad range of environmental conditions and to ensure sufficient biomass for reliable CARD-FISH detection. The sample was transferred into a sterile polypropylene bottle containing 24.3 mL of glyTE stock solution. The glyTE solution was prepared by mixing 20 mL of 100× TE buffer (pH 8.0), 60 mL of deionized water, and 100 mL of molecular-grade glycerol, followed by sterile filtration through a 0.1 µm pore-size membrane. After gently mixing the seawater with glyTE solution and incubation at ambient temperature for ~1 min, the sample was immediately stored at -80°C for subsequent analysis.

Prior to CARD-FISH, the sample was thawed at room temperature and gently mixed, then immediately fixed with freshly prepared 2% paraformaldehyde in the dark at 4°C for 12 hours. Subsequently, the sample was filtered through a 0.2 µm pore-size polycarbonate membrane (25 mm diameter; Millipore), rinsed three times with sterile water to remove residual glycerol, air-dried, and stored at -20°C until further processing. CARD-FISH was performed based on the protocol described by Eva Teira et al. [26] with minor modifications. Briefly, the membrane was embedded in low-gelling-point agarose, permeabilized with proteinase K (1 h, 37°C), and sectioned into small pieces for hybridization. Two-round hybridization was performed sequentially: (i) initial hybridization with an MAOA-specific horseradish peroxidase (HRP)-labeled probe and tyramide-Alexa488 signal amplification, followed by (ii) secondary hybridization with an HMN-specific HRP-labeled probe and tyramide-Cy3 amplification. All hybridizations were performed at 35°C for 12 h in hybridization buffer containing 20% (v/v) formamide. After each hybridization step, membrane pieces were washed with buffer containing 135 mM NaCl. Finally, hybridized samples were counterstained with DAPI, mounted in an antifading medium, and visualized using a laser scanning confocal microscope (LSM780 NLO, Carl Zeiss, Germany) equipped with a 63× oil immersion objective. To evaluate potential non-specific binding of the HMN-specific probe, an additional CARD-FISH experiment was conducted using 2 mL of pure culture of AOA strain *Nitrosopumilus maritimus* SCM1 grown in medium, following the previously described fixation and hybridization protocol. The results demonstrated a mean hybridization efficiency of 72.4% for the MAOA-specific probe, while the HMN-specific probe showed absolute specificity with no detectable cross-hybridization to SCM1 (Fig. S12). It should be noted that probe performance in pure cultures may not fully reflect its behavior in complex environmental samples.

To further assess potential non-specific binding of probes in complex environmental samples, two additional negative control experiments were performed using the nonspecific probe NON338 (5’-ACTCCTACGGGAGGCAGC-3’) [27]. All control experiments were conducted under the same hybridization conditions as the target probe experiments.

(i) Full negative control (probe NON338 in both hybridization steps): membrane sections were processed to the same two-round CARD-FISH procedure as described above, except that the nonspecific probe NON338 was used in both hybridization steps. Signal amplification was carried out using tyramide-Alexa488 in the first round and tyramide-Cy3 in the second round.

(ii) Negative control for nonspecific staining associated with MAOA-targeted labeling: membrane sections were first hybridized with the MAOA-specific HRP-labeled probe and signal amplification was performed using tyramide-Alexa488, followed by a second hybridization using the probe NON338 with tyramide-Cy3 amplification.

**Phylogenomic tree construction, molecular dating, and gene gain and loss**

Phylogenomic tree of 104 HMN genomes, 163 non-HMN Nitrososphaerota genomes, and 52 non-Nitrososphaerota archaeal genomes (Table S2), was constructed. Briefly, a multiple sequence alignment of 53 archaeal marker genes was generated using GTDB-Tk [9], then the maximum-likelihood phylogenomic tree was constructed using IQ-TREE v2.2.2.3 [28] under the best-fit model LG+F+I+R9 with 1000 bootstrap replicates. The phylogenetic divergence time of HMN was estimated using MCMCTree in PAML v4.9 [29] with seven calibration nodes, following the approach proposed by Yang et al. [30]. The parameters of the MCMCTree were set as “burnin = 1,000,000, sampfreq = 10, nsample = 500,000”. Seven nodes were calibrated with the following molecular calibration points: (ⅰ) The root node of the Archaea domain was assigned an old age range of 4.38–3.46 Ga [31–33] and a young age range of 3.8–2.7 Ga [34, 35]; (ⅱ) the independent origin of the *Thermoproteales*, *Sulfolobales*, and *Thermoplasma* lineages occurred after the GOE of 2.32 Ga [36]; (ⅲ) two clades, *Thermococcales* and *Halobacteriales*, originated after the first appearance of chitin-producing organisms, which have a maximum age constraint of 1.579 Ga [37, 38]; (ⅳ) horizontal gene transfer (HGT) from Viridiplantae to the ancestor of Nitrososphaerota Group I.1a and Group I.1b occurred between 1.487 and 0.75 Ga, based on which the divergence of Group I.1a and Group I.1b was set within this range [37, 39, 40]. MCMCTree was executed twice to confirm convergence (Fig. S3). To identify gene gain and loss events, the OGs of 104 HMN genomes and 163 non-HMN Nitrososphaerota genomes in the phylogenomic tree were generated using OrthoFinder v2.5.4 [16] with default parameters. The OG-table and tree file were input into the program COUNT [41] to reconstruct gene flux in the phylogenomic tree using the Dollo parsimony method.

**Potential gene exchange between HMN and other microorganisms**

The BLASTP-based similarities between the genes from 104 HMN genomes and those from 2,304 non-HMN MAGs from the *Tara Oceans* metagenomic dataset [13], as well as between the genes from 104 HMN genomes and those from 163 non-HMN Nitrososphaerota genomes (Table S2), were assessed using Diamond [19] with a threshold of 30% sequence identity, 50% coverage, and an e-value cut-off of 10^−20^. For obtaining the phylogenetic similarities between HMN genomes and other microorganisms, 31 conserved orthologous proteins [42] (Table S4) from each genome were identified using eggNOG-mapper v2.1.12 [17] with default parameters. These proteins were then concatenated into a single sequence to perform a Diamond BLASTP search against each other with a threshold of coverage > 3000 amino acid residues and an e-value cut-off of 10^−20^. By integrating the genome-level phylogenetic distances and gene-level similarity analyses, genes showing high sequence identity despite originating from phylogenetically distant genomes were considered as candidates for potential HGT events [43]. In addition, we also employed HGTector v2.0 [44], which is based on the distribution statistics of sequence homology search hits, by performing a Diamond BLASTP search against the NCBI-nr database with a threshold of 30% sequence identity, 50% coverage, and an e-value cut-off of 10^−20^, to identify HGT-derived genes in 104 HMN genomes and 163 non-HMN Nitrososphaerota genomes.

**Statistical analyses**

Differences between groups of data were tested using the non-parametric Mann-Whitney test because data were not always normally distributed. For the incubation experiment, however, a Student's t-test was used. Redundancy analysis (RDA) was conducted by integrating 202 metagenomes from 148 global samples (excluding metagenomes lacking environmental data) collected from surface water, the deep chlorophyll maximum layer, the mesopelagic zone, and the bathypelagic zone (Table S1 and S3) using the vegan package in *R*. The HMN genomes’ relative abundance (RPKM) matrix and the environmental dataset were used as response and explanatory matrices, respectively in the RDA. For samples with multiple metagenomes, the data is averaged and represented by a single dot in the RDA plot. The null hypothesis, that the community was independent of environmental parameters, was tested using constrained ordination with a Monte Carlo permutation test (999 permutations). For performing correlation analysis between the relative abundances of HMN and other marine microbes, a published operational taxonomic units (OTUs) table based on the 16S rRNA gene from the *Tara Oceans* metagenomics dataset and its corresponding taxonomic annotation were downloaded from https://ocean-microbiome.embl.de/companion.html.[45] Sixty-five samples are common between the 16S rRNA-based OTU table and the recruitment analysis mentioned above. Therefore, the top 1000 OTUs with the highest average relative abundance in these 65 samples were selected to perform Pearson correlation tests with the 104 HMN genomes, respectively. For correlation analysis between individual HMN and marine AOA (MAOA) genomes, the RPKM values of 63 MAOA genomes were obtained by recruiting reads from 272 global metagenomic libraries as described above, and Pearson correlation tests were conducted between RPKM values of the 104 HMN genomes and the 63 MAOA genomes using the vegan package in *R*. The regression analysis between total HMN and MAOA communities were performed using log-transformed sums of RPKM values of 104 HMN and 63 MAOA genomes.

**Supplementary results**

**Metabolic adaptations of heterotrophic marine Nitrososphaerota (HMN) ecotypes to niches**

The shallow ecotype of HMN (SEH) exhibits a distinct genetic repertoire, including uniquely present or highly expressed genes involved in DNA replication and repair, such as DNA polymerase subunits (*dnaN*, and *holA*), transcription-repair coupling factor (*mfd*), mismatch repair-related exodeoxyribonuclease (*xseA*), and DNA adenine methylase (*dam*), compared to the deep ecotype (DEH) (Fig. S14). The acquisition of these genes may have endowed SEH with the ability to resist light-induced DNA damage, a capacity that DEH does not share. This case is similar to marine ammonia-oxidizing (MAOA), in which the water column group A (WCA) contains ultraviolet light-resistance *uvr* genes, while the water column group B (WCB) has lost them [46]. Indeed, *uvrD* was also among the top expressed genes of SEH in the epipelagic zone, although it exists in both SEH and DEH genomes (Fig. S14). Similar to the inhibition of AOA activity by ROS [47, 48], DEH may also be sensitive to ROS-induced cell damage due to a lack of the catalase gene. Although HMN genomes encode a nickel superoxide dismutase (*sodN*)—similar to the Fe-Mn family superoxide dismutase found in marine AOA that catalyzes superoxide disproportionation to peroxide and molecular oxygen (Fig. S14)—this single enzyme may still be insufficient to prevent cell oxidative damage. However, SEH may enhance ROS resistance by uniquely encoding glutathione peroxidase (*gpx*), an enzyme that reduces hydrogen peroxide and organic hydroperoxides to water/alcohols using reduced glutathione [49], unlike DEH (Fig. S14). The most actively expressed unique KEGG orthology group in SEH was associated with *ppa*, encoding an inorganic pyrophosphatase involved in the hydrolysis of inorganic phosphate polymers (Fig. S14). This enzyme plays a crucial role in utilizing intracellular phosphate reserves under phosphorus starvation conditions [50–52], suggesting that SEH has a better adaptation to phosphorus-limited conditions than DEH in the upper ocean. These results suggest that DEH and marine AOA share similar physiological motivations for inhabiting the deep sea, specifically to avoid the destructive effects of light and ROS on cells, as well as to escape competition with phytoplankton and bacterioplankton for essential nutrients in the photic zone. Furthermore, DEH may have enhanced its cold resistance by actively expressing temperature-sensitive proteins such as CshA and eEF2 (Fig. S14). These proteins assist in the folding of RNA and translation under cold-induced stress [53–55]. Overall, these genomic features indicate distinct ecological adaptation strategies between SEH and DEH, particularly in terms of stress tolerance (e.g., UV radiation, ROS, and temperature) and resource utilization (e.g., phosphorus acquisition). These functional differences provide mechanistic support for the ecological differentiation observed between shallow and deep HMN ecotypes.

**References**

1. Massana R, Murray AE, Preston CM, DeLong EF. Vertical distribution and phylogenetic characterization of marine planktonic Archaea in the Santa Barbara Channel. *Appl Environ Microbiol* 1997;**63**:50-56. 10.1128/aem.63.1.50-56.1997.
2. Chen S, Zhou Y, Chen Y, Gu J. fastp: an ultra-fast all-in-one FASTQ preprocessor. *Bioinformatics* 2018;**34**:i884-i890. 10.1093/bioinformatics/bty560.
3. Li D, Liu CM, Luo R, Sadakane K, Lam TW. MEGAHIT: an ultra-fast single-node solution for large and complex metagenomics assembly via succinct de Bruijn graph. *Bioinformatics* 2015;**31**:1674-1676. 10.1093/bioinformatics/btv033.
4. Alneberg J, Bjarnason BS, de Bruijn I, Schirmer M, Quick J, Ijaz UZ. *et al*. Binning metagenomic contigs by coverage and composition. *Nat Methods* 2014;**11**:1144-1146. 10.1038/nmeth.3103.
5. Kang DD, Li F, Kirton E, Thomas A, Egan R, An H. *et al*. MetaBAT 2: an adaptive binning algorithm for robust and efficient genome reconstruction from metagenome assemblies. *PeerJ* 2019;**7**:e7359. 10.7717/peerj.7359.
6. Wu YW, Simmons BA, Singer SW. MaxBin 2.0: an automated binning algorithm to recover genomes from multiple metagenomic datasets. *Bioinformatics* 2016;**32**:605-607. 10.1093/bioinformatics/btv638.
7. Olm MR, Brown CT, Brooks B, Banfield JF. dRep: a tool for fast and accurate genomic comparisons that enables improved genome recovery from metagenomes through de-replication. *ISME J* 2017;**11**:2864-2868. 10.1038/ismej.2017.126.
8. Parks DH, Imelfort M, Skennerton CT, Hugenholtz P, Tyson GW. CheckM: assessing the quality of microbial genomes recovered from isolates, single cells, and metagenomes. *Genome Res* 2015;**25**:1043-1055. 10.1101/gr.186072.114.
9. Chaumeil PA, Mussig AJ, Hugenholtz P, Parks DH. GTDB-Tk: a toolkit to classify genomes with the Genome Taxonomy Database. *Bioinformatics* 2020;**36**:1925-1927. 10.1093/bioinformatics/btz848.
10. Reji L, Francis CA. Metagenome-assembled genomes reveal unique metabolic adaptations of a basal marine Thaumarchaeota lineage. *ISME J* 2020;**14**:2105-2115. 10.1038/s41396-020-0675-6.
11. Kopylova E, Noé L, Touzet H. SortMeRNA: fast and accurate filtering of ribosomal RNAs in metatranscriptomic data. *Bioinformatics* 2012;**28**:3211-3217. 10.1093/bioinformatics/bts611.
12. Langmead B, Salzberg SL. Fast gapped-read alignment with Bowtie 2. *Nat Methods* 2012;**9**:357-359. 10.1038/nmeth.1923.
13. Tully BJ, Graham ED, Heidelberg JF. The reconstruction of 2,631 draft metagenome-assembled genomes from the global oceans. *Sci Data* 2018;**5**:170203. 10.1038/sdata.2017.203.
14. Hyatt D, Chen GL, LoCascio PF, Land ML, Larimer FW, Hauser LJ. Prodigal: prokaryotic gene recognition and translation initiation site identification. *BMC Bioinformatics* 2010;**11**:119. 10.1186/1471-2105-11-119.
15. Mende DR, Bryant JA, Aylward FO, Eppley JM, Nielsen T, Karl DM. *et al*. Environmental drivers of a microbial genomic transition zone in the ocean’s interior. *Nat Microbiol* 2017;**2**:1367-1373. 10.1038/s41564-017-0008-3.
16. Emms DM, Kelly S. OrthoFinder: phylogenetic orthology inference for comparative genomics. *Genome Biol* 2019;**20**:238. 10.1186/s13059-019-1832-y.
17. Cantalapiedra CP, Hernández-Plaza A, Letunic I, Bork P, Huerta-Cepas J. eggNOG-mapper v2: functional annotation, orthology assignments, and domain prediction at the metagenomic scale. *Mol Biol Evol* 2021;**38**:5825-5829. 10.1093/molbev/msab293.
18. Kanehisa M, Goto S. KEGG: Kyoto Encyclopedia of Genes and Genomes. *Nucleic Acids Res* 2000;**28**:27-30. 10.1093/nar/28.1.27.
19. Buchfink B, Xie C, Huson DH. Fast and sensitive protein alignment using DIAMOND. *Nat Methods* 2015;**12**:59-60. 10.1038/nmeth.3176.
20. Makarova KS, Wolf YI, Koonin EV. Archaeal clusters of orthologous genes (arCOGs): an update and application for analysis of shared features between Thermococcales, Methanococcales, and Methanobacteriales. *Life* 2015;**5**:818-840. 10.3390/life5010818.
21. Yin Y, Mao X, Yang J, Chen X, Mao F, Xu Y. dbCAN: a web resource for automated carbohydrate-active enzyme annotation. *Nucleic Acids Res* 2012;**40**:W445-W451. 10.1093/nar/gks479.
22. Rawlings ND, Barrett AJ, Thomas PD, Huang X, Bateman A, Finn RD. The MEROPS database of proteolytic enzymes, their substrates and inhibitors in 2017 and a comparison with peptidases in the PANTHER database. *Nucleic Acids Res* 2018;**46**:D624-D632. 10.1093/nar/gkx1134.
23. Teufel F, Almagro Armenteros JJ, Johansen AR, Gíslason MH, Pihl SI, Tsirigos KD. *et al*. SignalP 6.0 predicts all five types of signal peptides using protein language models. *Nat Biotechnol* 2022;**40**:1023-1025. 10.1038/s41587-021-01156-3.
24. Bayer B, Liu S, Louie K, Northen TR, Wagner M, Daims H. *et al*. Metabolite release by nitrifiers facilitates metabolic interactions in the ocean. *ISME J* 2024;**18**:wrae172. 10.1093/ismejo/wrae172.
25. Katoh K, Misawa K, Kuma K, Miyata T. MAFFT: a novel method for rapid multiple sequence alignment based on fast Fourier transform. *Nucleic Acids Res* 2002;30:3059-3066. 10.1093/nar/gkf436.
26. Teira E, Reinthaler T, Pernthaler A, Pernthaler J, Herndl GJ. Combining catalyzed reporter deposition-fluorescence in situ hybridization and microautoradiography to detect substrate utilization by bacteria and archaea in the deep ocean. *Appl Environ Microbiol* 2004;**70**:4411-4414. 10.1128/AEM.70.7.4411-4414.2004.
27. Wallner G, Amann R, Beisker W. Optimizing fluorescent in situ hybridization with rRNA-targeted oligonucleotide probes for flow cytometric identification of microorganisms. *Cytometry* 1993;14:136-143. 10.1002/cyto.990140205.
28. Minh BQ, Schmidt HA, Chernomor O, Schrempf D, Woodhams MD, von Haeseler A. *et al*. IQ-TREE 2: new models and efficient methods for phylogenetic inference in the genomic era. *Mol Biol Evol* 2020;**37**:1530-1534. 10.1093/molbev/msaa015.
29. Yang Z. PAML 4: phylogenetic analysis by maximum likelihood. *Mol Biol Evol* 2007;**24**:1586-1591. 10.1093/molbev/msm088.
30. Yang Y, Zhang C, Lenton TM, Yan X, Zhu M, Zhou M. *et al*. The evolution pathway of ammonia-oxidizing archaea shaped by major geological events. *Mol Biol Evol* 2021;**38**:3637-3648. 10.1093/molbev/msab129.
31. Ueno Y, Johnson MS, Danielache SO, Eskebjerg C, Pandey A, Yoshida N. Geological sulfur isotopes indicate elevated OCS in the Archean atmosphere, solving faint young sun paradox. *Proc Natl Acad Sci USA* 2009;**106**:14784-14789. 10.1073/pnas.0903518106.
32. Valley JW, Cavosie AJ, Ushikubo T, Reinhard DA, Lawrence DF, Larson DJ. *et al*. Hadean age for a post-magma-ocean zircon confirmed by atom-probe tomography. *Nat Geosci* 2014;**7**:219-223. 10.1038/ngeo2075.
33. Wolfe JM, Fournier GP. Horizontal gene transfer constrains the timing of methanogen evolution. *Nat Ecol Evol* 2018;**2**:897-903. 10.1038/s41559-018-0513-7.
34. Blank CE. Not so old Archaea–the antiquity of biogeochemical processes in the archaeal domain of life. *Geobiology* 2009;**7**:495-514. 10.1111/j.1472-4669.2009.00219.x.
35. Blank CE. An expansion of age constraints for microbial clades that lack a conventional fossil record using phylogenomic dating. *J Mol Evol* 2011;**73**:188-208. 10.1007/s00239-011-9467-y.
36. Bekker A, Holland HD, Wang PL, Rumble D, Stein HJ, Hannah JL. *et al*. Dating the rise of atmospheric oxygen. *Nature* 2004;**427**:117-120. 10.1038/nature02260.
37. Parfrey LW, Lahr DJG, Knoll AH, Katz LA. Estimating the timing of early eukaryotic diversification with multigene molecular clocks. *Proc Natl Acad Sci USA* 2011;**108**:13624-13629. 10.1073/pnas.1110633108.
38. Eme L, Sharpe SC, Brown MW, Roger AJ. On the age of eukaryotes: evaluating evidence from fossils and molecular clocks. *Cold Spring Harbor Perspect Biol* 2014;**6**:a016139. 10.1101/cshperspect.a016139.
39. Douzery EJP, Snell EA, Bapteste E, Delsuc F, Philippe H. The timing of eukaryotic evolution: Does a relaxed molecular clock reconcile proteins and fossils? *Proc Natl Acad Sci USA* 2004;**101**:15386-15391. 10.1073/pnas.0403984101.
40. Petitjean C, Moreira D, López-García P, Brochier-Armanet C. Horizontal gene transfer of a chloroplast DnaJ-Fer protein to Thaumarchaeota and the evolutionary history of the DnaK chaperone system in Archaea. *BMC Evol Biol* 2012;**12**:226. 10.1186/1471-2148-12-226.
41. Csűös M. Count: evolutionary analysis of phylogenetic profiles with parsimony and likelihood. *Bioinformatics* 2010;**26**:1910-1912. 10.1093/bioinformatics/btq315.
42. Ciccarelli FD, Doerks T, von Mering C, Creevey CJ, Snel B, Bork P. Toward automatic reconstruction of a highly resolved tree of life. *Science* 2006;**311**:1283-1287. 10.1126/science.1123061.
43. Kurland CG, Canback B, Berg OG. Horizontal gene transfer: A critical view. *Proc Natl Acad Sci USA* 2003;**100**:9658-9662. 10.1073/pnas.1632870100.
44. Zhu Q, Kosoy M, Dittmar K. HGTector: an automated method facilitating genome-wide discovery of putative horizontal gene transfers. *BMC Genomics* 2014;**15**:717. 10.1186/1471-2164-15-717.
45. Sunagawa S, Coelho LP, Chaffron S, Kultima JR, Labadie K, Salazar G. *et al*. Structure and function of the global ocean microbiome. *Science* 2015;**348**:1261359. 10.1126/science.1261359.
46. Ren M, Feng X, Huang Y, Wang H, Hu Z, Clingenpeel S. *et al*. Phylogenomics suggests oxygen availability as a driving force in Thaumarchaeota evolution. *ISME J* 2019;**13**:2150-2161. 10.1038/s41396-019-0418-8.
47. Tolar BB, Powers LC, Miller WL, Wallsgrove NJ, Popp BN, Hollibaugh JT. Ammonia oxidation in the ocean can be inhibited by nanomolar concentrations of hydrogen peroxide. *Front Mar Sci* 2016;**3**:1-16. 10.3389/fmars.2016.00237.
48. Wang H, Li P, Liu X, Zhang J, Stein LY, Gu JD. An overlooked influence of reactive oxygen species on ammonia-oxidizing microbial communities in redox-fluctuating aquifers. *Water Res* 2023;**233**:119734. 10.1016/j.watres.2023.119734.
49. Margis R, Dunand C, Teixeira FK, Margis-Pinheiro M. Glutathione peroxidase family – an evolutionary overview. *FEBS J* 2008;**275**:3959-3970. 10.1111/j.1742-4658.2008.06542.x.
50. Gómez-Garcı́a MR, Losada M, Serrano A. Concurrent transcriptional activation of ppa and ppx genes by phosphate deprivation in the cyanobacterium Synechocystis sp. strain PCC 6803. *Biochem Biophys Res Commun* 2003;**302**:601-609. 10.1016/S0006-291X(03)00162-1.
51. Peimbert M, Alcaraz LD, Bonilla-Rosso G, Olmedo-Alvarez G, García-Oliva F, Segovia L. *et al*. Comparative metagenomics of two microbial mats at cuatro ciénegas basin I: ancient lessons on how to cope with an environment under severe nutrient stress. *Astrobiology* 2012;**12**:648-658. 10.1089/ast.2011.0694.
52. Pereira N, Shilova IN, Zehr JP. Molecular markers define progressing stages of phosphorus limitation in the nitrogen-fixing cyanobacterium, Crocosphaera. *J Phycol* 2016;**52**:274-282. 10.1111/jpy.12396.
53. Thomas T, Cavicchioli R. Cold adaptation of archaeal elongation factor 2 (EF-2) proteins. *Curr Protein Pept Sci* 2002;**3**:223-230. 10.2174/1389203024605359.
54. Cavicchioli R. Cold-adapted archaea. *Nat Rev Microbiol* 2006;**4**:331-343. 10.1038/nrmicro1390.
55. Hunger K, Beckering CL, Wiegeshoff F, Graumann PL, Marahiel MA. Cold-induced putative DEAD Box RNA helicases CshA and CshB are essential for cold adaptation and interact with cold shock protein B in *Bacillus subtilis*. *J Bacteriol* 2006;**188**:240-248. 10.1128/jb.188.1.240-248.2006.


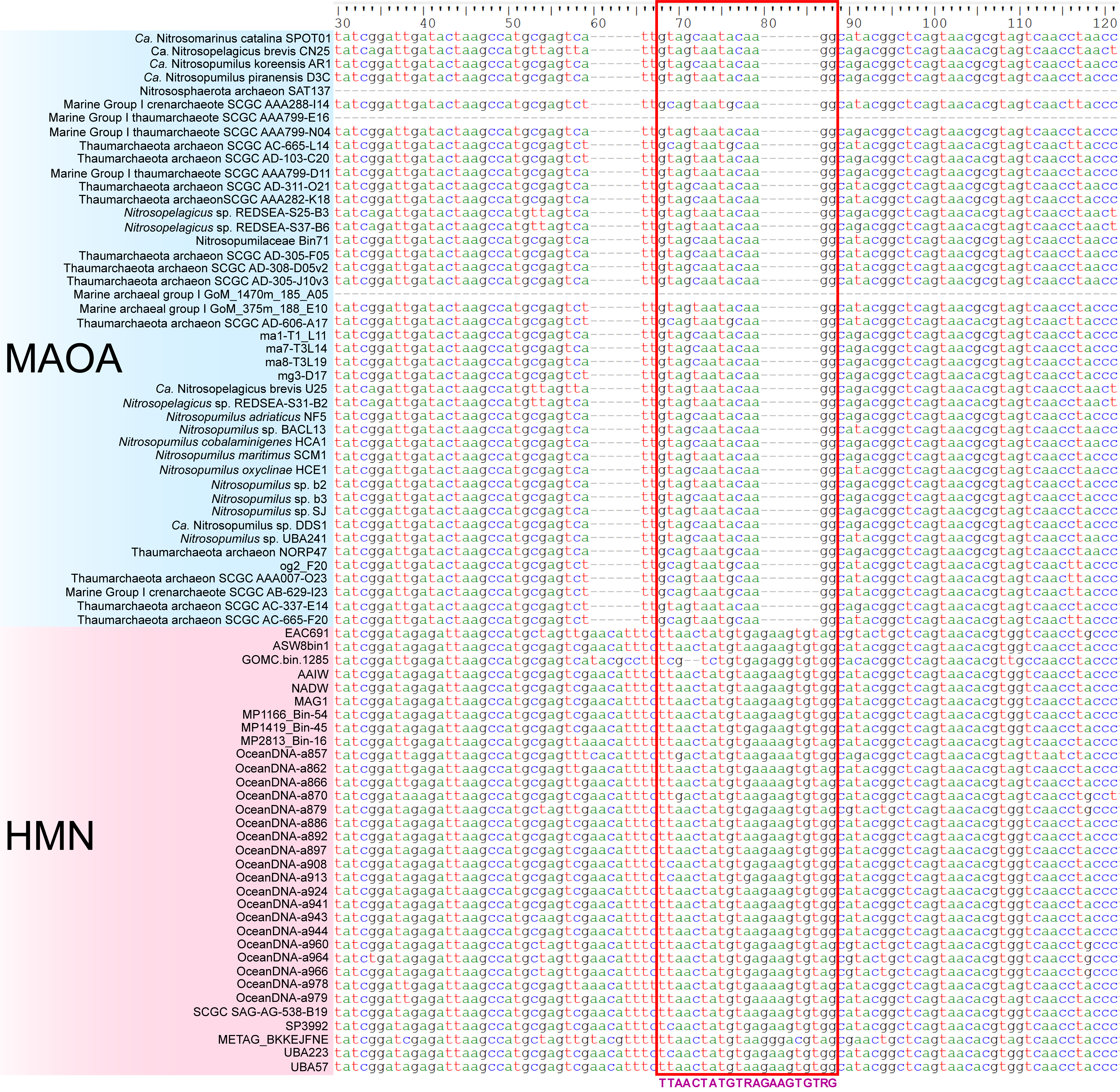


**Fig. S1.** **Conserved sequence region targeted for heterotrophic marine Nitrososphaerota (HMN)-specific 16S rRNA gene probe design.** MAOA, marine ammonia-oxidizing archaea.


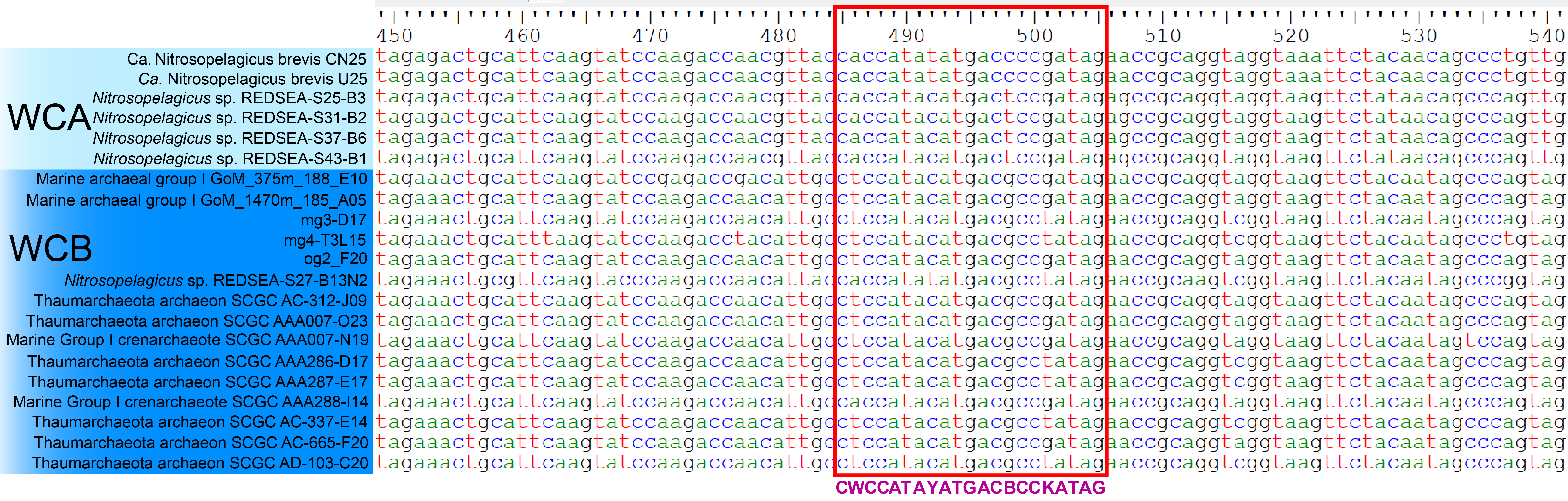


**Fig. S2.** **Conserved sequence region targeted for marine ammonia-oxidizing archaea (MAOA)-specific *amoA* gene probe design.** WCA, water column group A; WCB, water column group B.





**Fig. S3. Fitting a function using the temporal nodes from two runs in MCMCTree.**


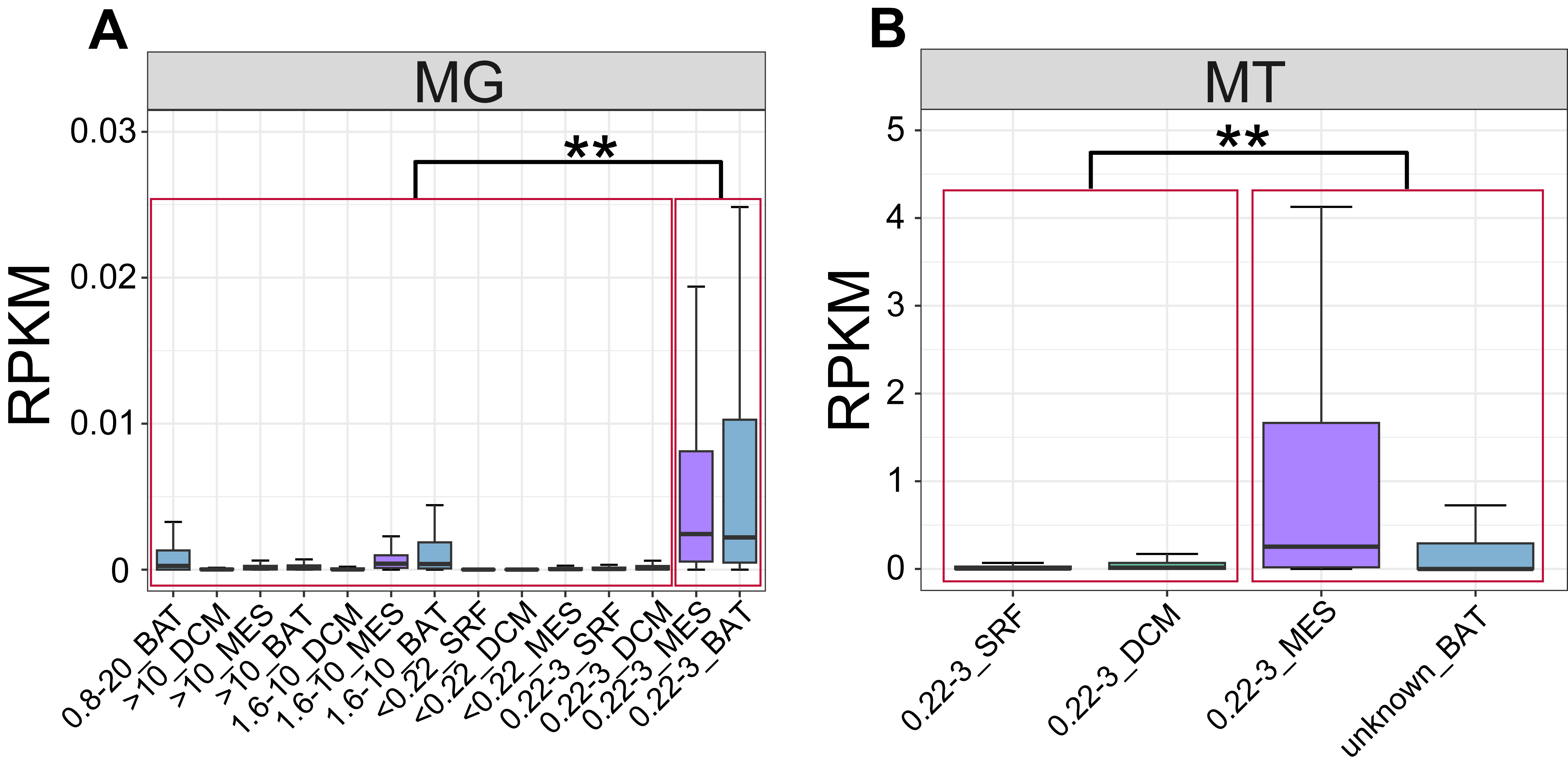


**Fig. S4.** **Overall distribution of RPKMs (see Methods) for heterotrophic marine Nitrososphaerota (HMN) genomes in different (A) metagenomic and (B) metatranscriptomic datasets.** The box represents the median and interquartile range, with whiskers extending to 1.5 times the interquartile range from the lower and upper quartiles. MG, metagenomic datasets; MT, metatranscriptomic datasets. Abbreviations indicating the sources of MG and MT samples are as follows: “<0.22 (<0.22 μm)/0.22–3 (0.22–3 μm)/0.8–20 (0.8–20 μm)/1.6–10 (1.6–10 μm)/>10 (>10 μm)/unknown (no data)”_“SRF (surface water)/DCM (deep chlorophyll maximum layer)/MES (mesopelagic zone)/BAT (bathypelagic zone)”. For MG datasets, the size fraction of 0.22–3 μm includes samples with sizes of 0.22–0.8 μm, 0.3–0.8 μm, 0.22–1.6 μm, 0.3–1.6 μm, 0.22–3 μm, and 0.3–3 μm. For MT datasets, the size fraction of 0.22–3 μm includes samples with sizes of 0.22–1.6 μm and 0.22–3 μm. ***P* < 0.01, Mann-Whitney test.





**Fig. S5.** Global distribution of heterotrophic marine Nitrososphaerota (HMN). (A) Heatmap of log-transformed RPKM values (see Methods) for HMN genomes across 272 global ocean metagenomes and (B) 156 metatranscriptomes. Average linkage hierarchical clustering based on Bray-Curtis dissimilarity distances of range-standardized RPKMs is shown on the right. Sample sources, depths, and size fractions are indicated by different colors. SCS, South China Sea; SRF, surface water; DCM, deep chlorophyll maximum layer; MIX, mixed layer; EUP, euphotic zone; MES, mesopelagic zone; BAT, bathypelagic zone. Genomes shaded in green represent SEH, while those shaded in blue represent DEH.


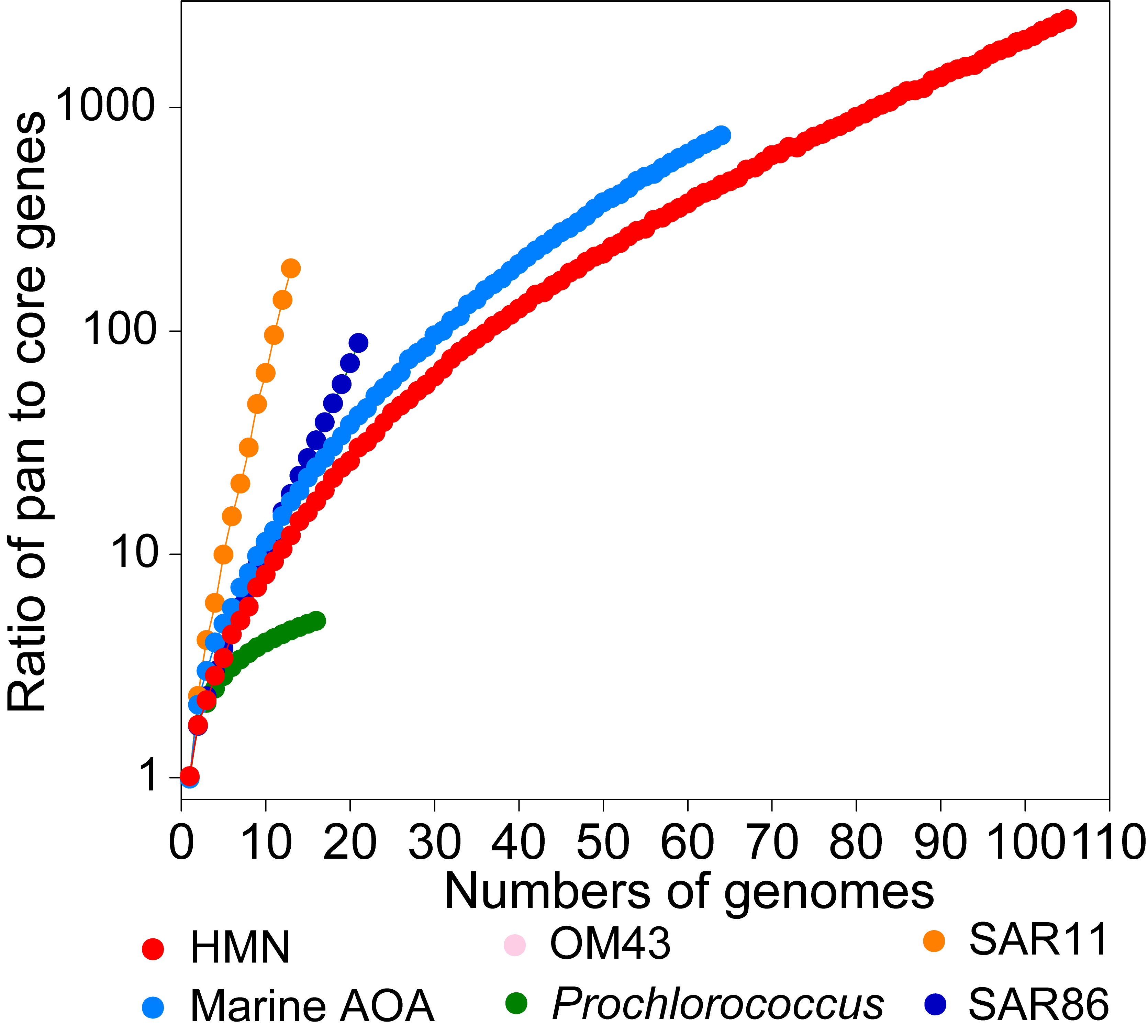


**Fig. S6. Ratio of pan-genome size to core-genome size as a function of sequentially added genomes in HMN and five model streamlined marine microbes.** For each step n (number of genomes added), a random subsampling of n genomes was repeated 1,000 times from the total genome pool to calculate the average pan-to-core genome size ratio. AOA, ammonia-oxidizing archaea.


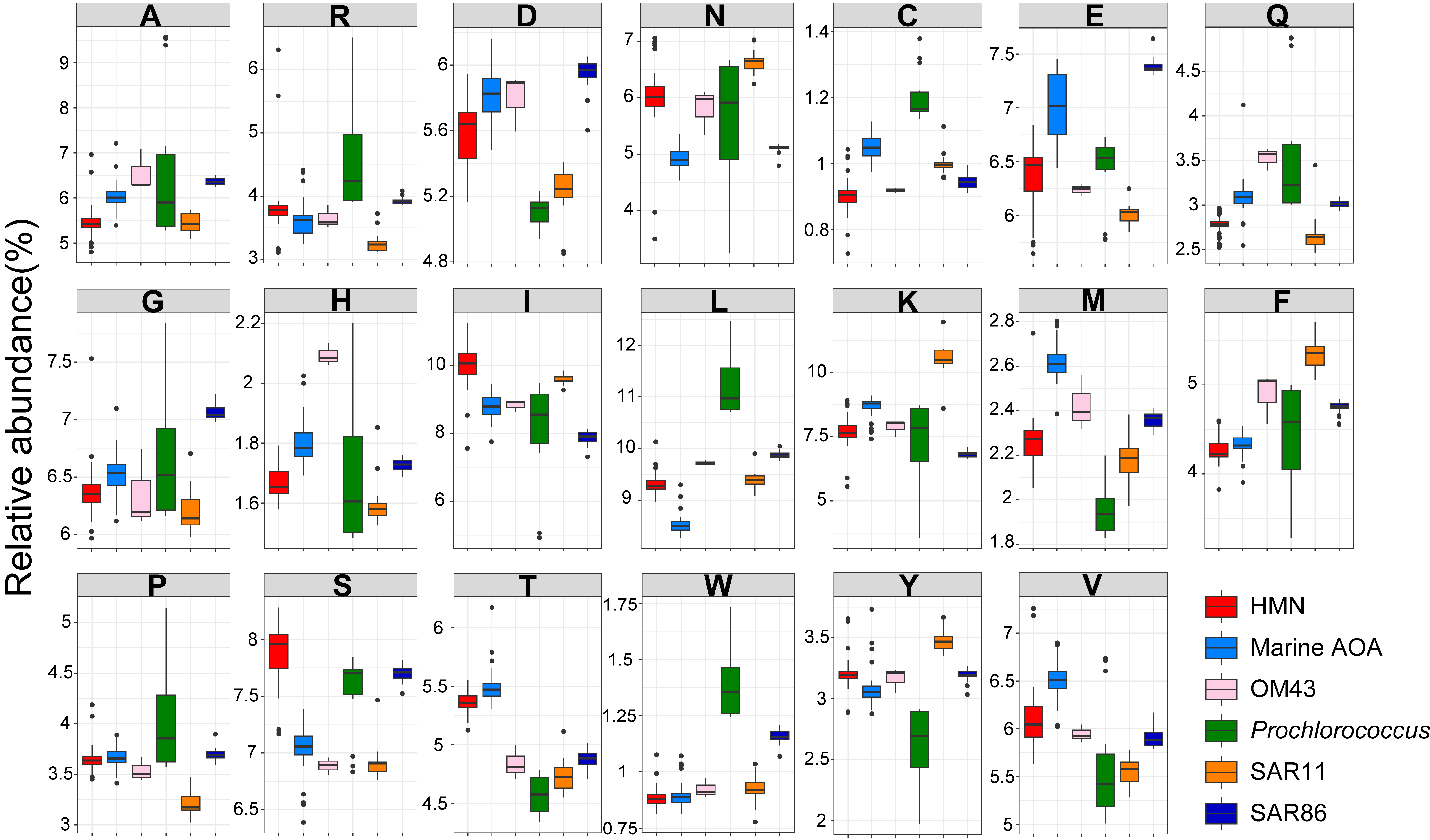


**Fig. S7. Relative abundance of each amino acid in the encoded proteins of HMN and five model streamlined marine microbes.** AOA, ammonia-oxidizing archaea; A, Alanine; R, Arginine; N, Asparagine; D, Aspartic acid; C, Cysteine; Q, Glutamine; E, Glutamic acid; G, Glycine; H, Histidine; I, Isoleucine; L, Leucine; K, Lysine; M, Methionine; F, Phenylalanine; P, Proline; S, Serine; T, Threonine; W, Tryptophan; Y, Tyrosine; V, Valine.


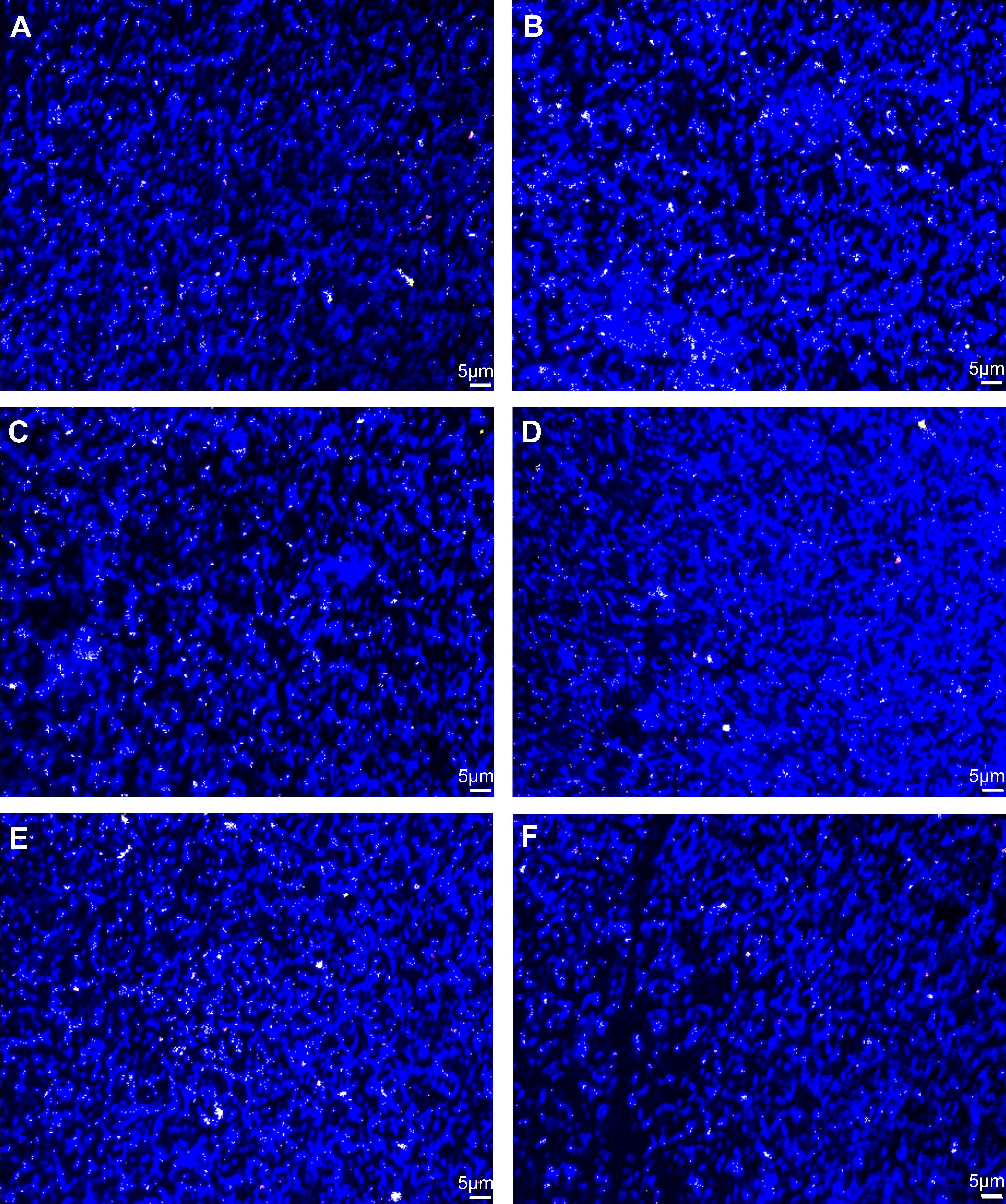


**Fig. S8. Catalyzed reporter deposition fluorescence in situ hybridization (CARD-FISH) of a Western Pacific Ocean seawater mixed sample.** The image shows merged channels for DAPI (blue, DNA stain), marine ammonia-oxidizing archaea (MAOA)-specific probes (yellow), and heterotrophic marine Nitrososphaerota (HMN)-specific probes (magenta). Panels A‒F are representative microscopic fields of view. Note: The seawater sample was concentrated (from an initial volume of 243 mL) prior to CARD-FISH to obtain a sufficient number of HMN cells for statistical analysis. This concentration step results in the densely packed and layered appearance of DAPI signals in the images.


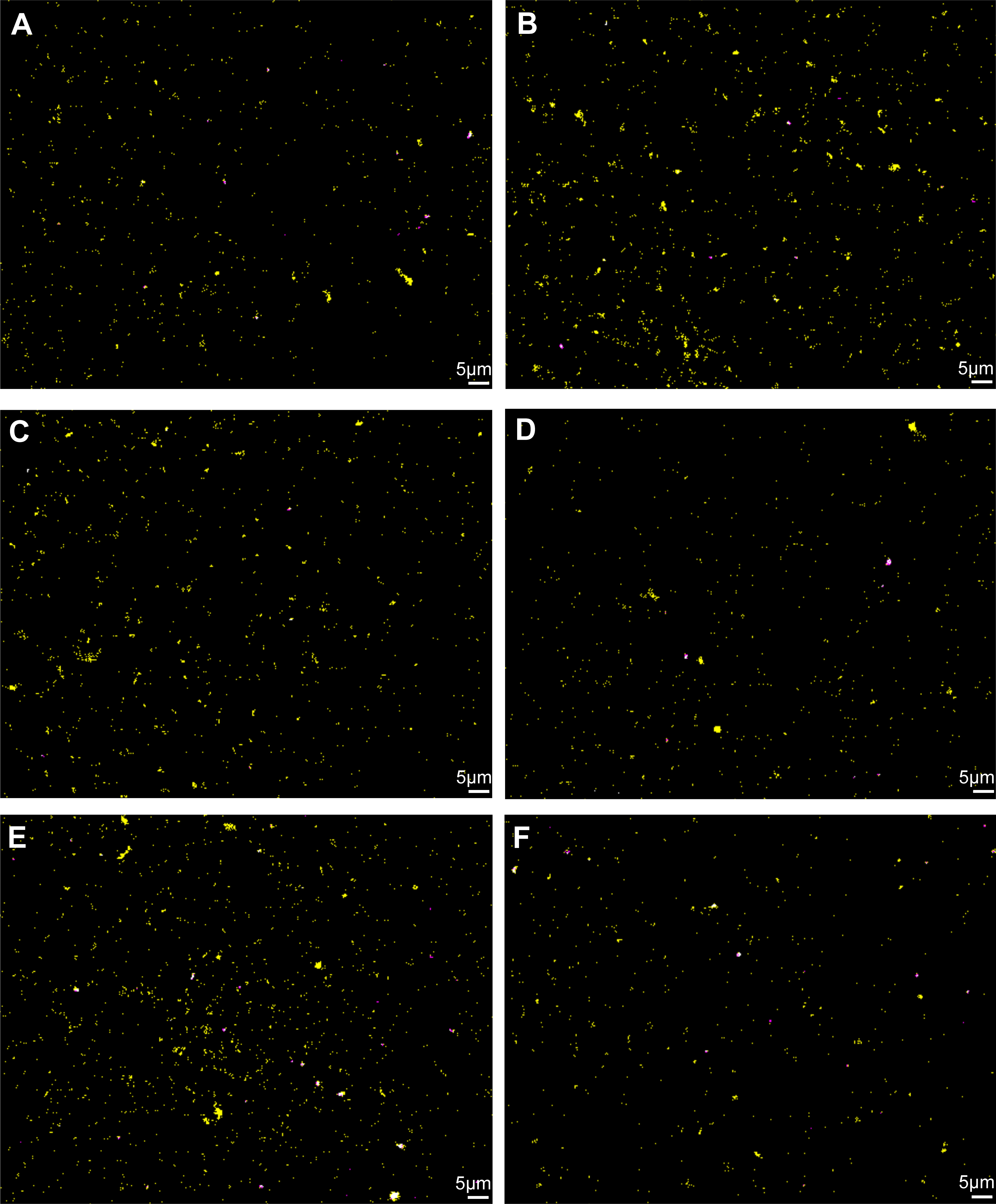


**Fig. S9.** **Catalyzed reporter deposition fluorescence in situ hybridization (CARD-FISH) of a Western Pacific Ocean seawater mixed sample hybridized with marine ammonia-oxidizing archaea (MAOA)-specific (yellow) and heterotrophic marine Nitrososphaerota (HMN)-specific (magenta) probes.** Panels A‒F show different microscopic fields of view; each corresponds to the identically labeled field in Figure S8.


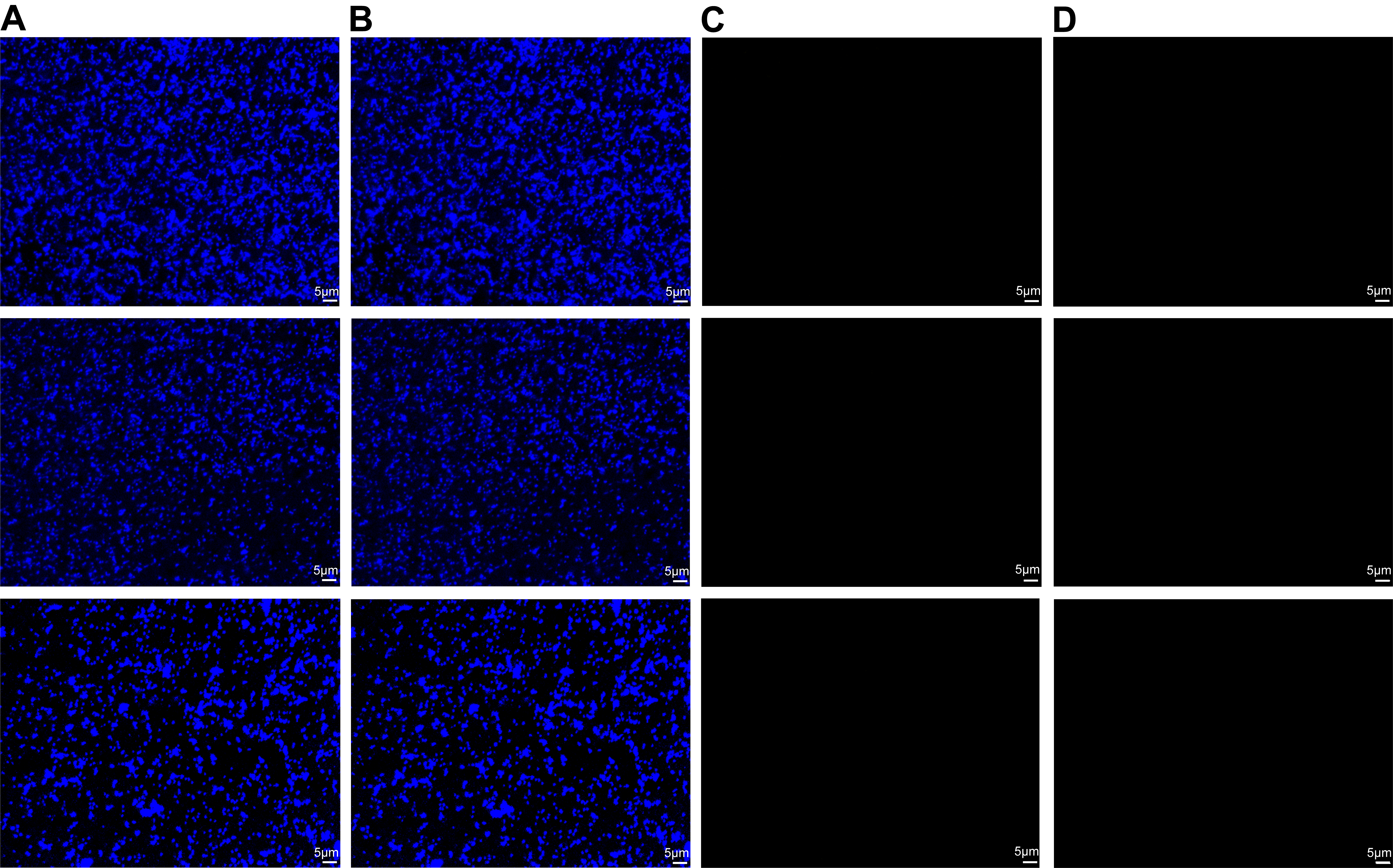


**Fig. S10. Negative control for catalyzed reporter deposition fluorescence in situ hybridization (CARD-FISH) using the nonspecific probe NON338 of a Western Pacific Ocean seawater mixed sample.** CARD-FISH was performed as a two-round procedure using the nonspecific probe NON338 in both hybridization steps. Column (A) displays merged images: DAPI staining (blue), tyramide-Alexa488 signal (yellow), and tyramide-Cy3 signal (magenta). Column (B) shows DAPI staining (blue). Column (C) shows the tyramide-Alexa488 signal (yellow). Column (D) shows the tyramide-Cy3 signal (magenta). No fluorescence signal was observed in either tyramide-Alexa488 or tyramide-Cy3 channels. Images in the same row represent the same microscopic field of view.


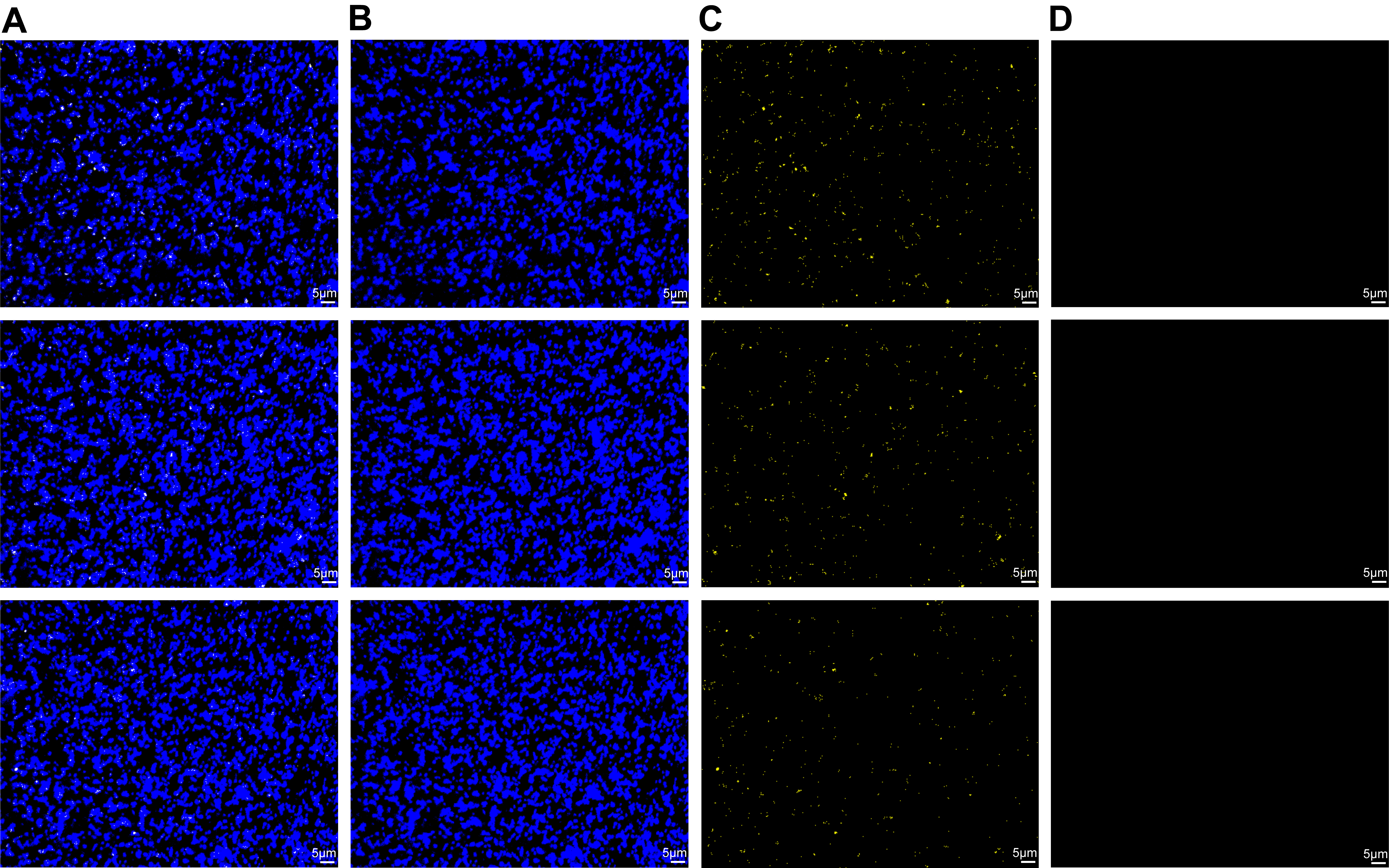


**Fig. S11.** **Negative control for MAOA-targeted catalyzed reporter deposition fluorescence in situ hybridization (CARD-FISH) in a Western Pacific Ocean seawater mixed sample.** Column (A) displays merged images: DAPI staining (blue), tyramide-Alexa488 signal (yellow), and tyramide-Cy3 signal (magenta). Column (B) shows DAPI staining (blue). Column (C) shows the tyramide-Alexa488 signal (yellow). Column (D) shows the tyramide-Cy3 signal (magenta). No fluorescence signal was observed in tyramide-Cy3 channels. Images in the same row represent the same microscopic field of view.


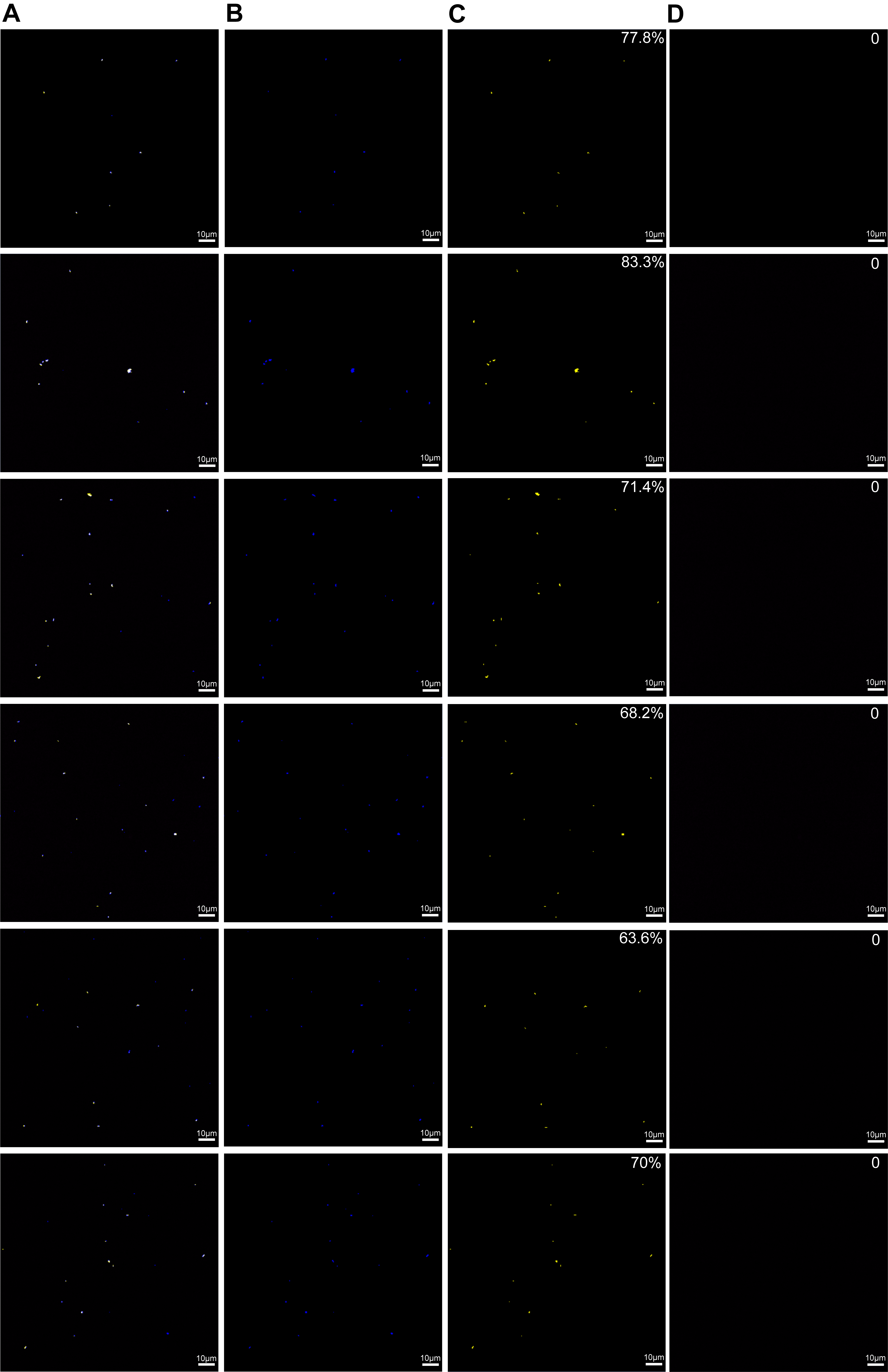


**Fig. S12.** **Evaluation of potential non-specific binding of heterotrophic marine Nitrososphaerota (HMN) probe using the pure culture Nitrosopumilus maritimus SCM1.** Column (**A**) displays catalyzed reporter deposition fluorescence in situ hybridization (CARD-FISH) results of the pure AOA culture *Nitrosopumilus maritimus* SCM1: DAPI stain (blue); MAOA-specific probes (yellow); and heterotrophic marine Nitrososphaerota (HMN)-specific probes (magenta; no signal observed). Column (**B**) shows DAPI staining (blue) of SCM1. Column (**C**) presents CARD-FISH with solely the MAOA-specific *amoA* probe (yellow), with hybridization efficiency percentage indicated in the upper right corner. Column (**D**) shows CARD-FISH with only the HMN-specific 16S rRNA probe (magenta; no signal detected, indicated by “0” in top-right corner). Images in the same row represent the same microscopic field of view.


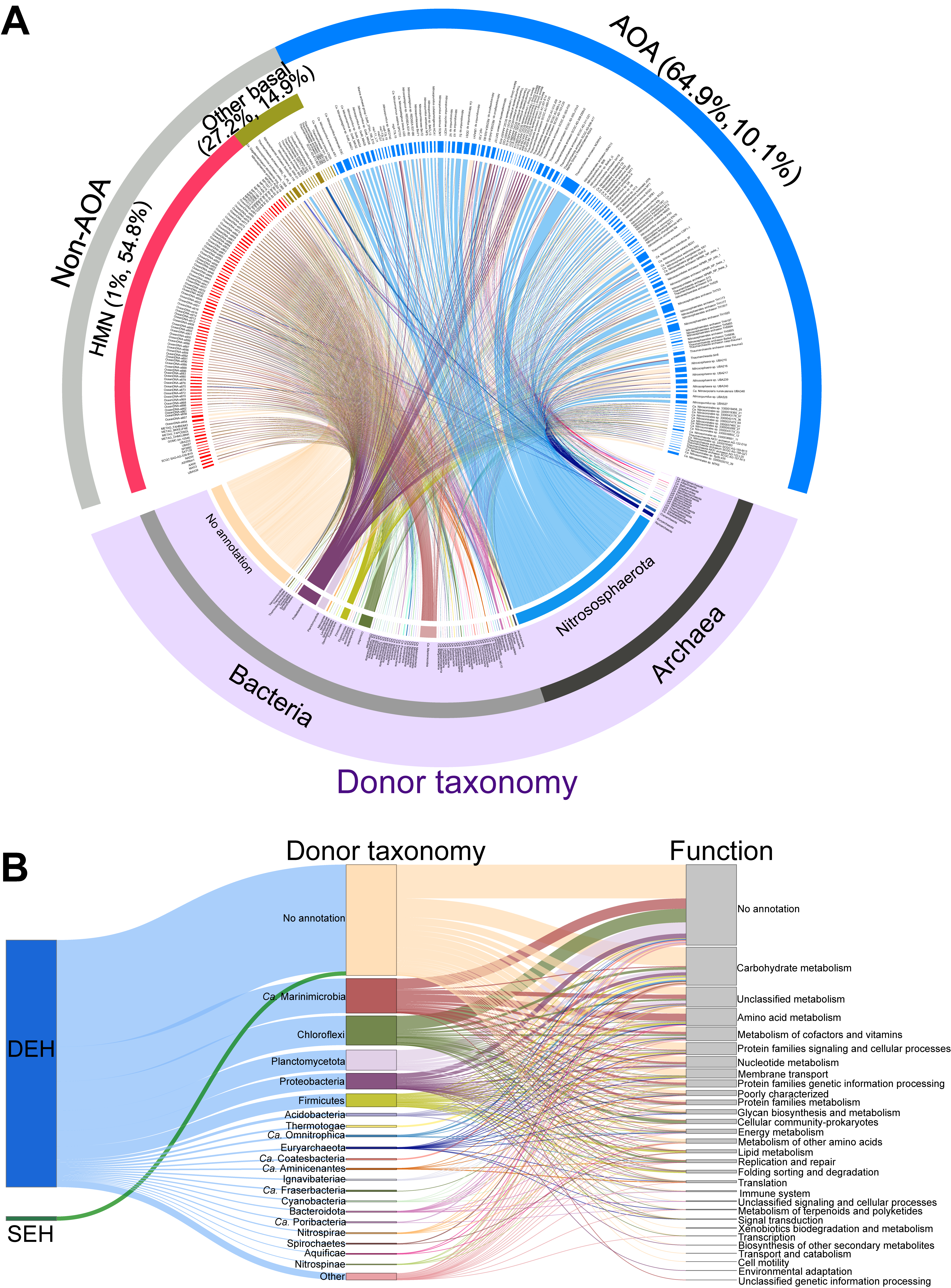


**Fig. S13.** **Horizontal gene transfer events (HGTs) detected in Nitrososphaerota using HGTector v2.0.** (**A**) HGTs predicted in 253 Nitrososphaerota genomes (upper part) from other bacteria and archaea (lower part). The lines represent potential gene flows. The thickness of each line is proportional to the number of putatively transferred genes. Proportions in parentheses indicate the percentage of transferred genes from archaea (left) and the proportion of transferred genes from bacteria (right). (**B**) Donor taxonomy and function of predicted transferred genes in the genomes of heterotrophic marine Nitrososphaerota (HMN) from the shallow ecotype (SEH) and deep ecotype (DEH), based on annotations from NCBI-nr and KEGG databases.





**Fig. S14.** **Metabolic reconstruction overview of heterotrophic marine Nitrososphaerota (HMN).** Solid arrows represent confidently annotated genes, while dashed arrows and crosses (x) denote either unidentified genes or genes with extremely low prevalence probability below estimated cutoff (defined in Methods). The transcriptional levels of key genes are presented as RPKM values (see Methods). The accompanying pie chart displays the detection frequency of each gene across all analyzed genomes of the shallow (SEH) and deep ecotypes (DEH) of HMN, presented separately. Key genes discussed in the main text are highlighted in purple columns and labeled with gene abbreviations (explained in Table S6). Genes involved in horizontal gene transfer are marked with asterisks (*).
